# Evolution, Mode of Transmission, and Mutational Landscape of Newly Emerging SARS-CoV-2 Variants

**DOI:** 10.1128/mBio.01140-21

**Published:** 2021-08-31

**Authors:** Chiranjib Chakraborty, Ashish Ranjan Sharma, Manojit Bhattacharya, Govindasamy Agoramoorthy, Sang-Soo Lee

**Affiliations:** a Department of Biotechnology, School of Life Science and Biotechnology, Adamas University, Kolkata, West Bengal, India; b Institute for Skeletal Aging & Orthopedic Surgery, Hallym University-Chuncheon Sacred Heart Hospital, Chuncheon-si, Gangwon-do, Republic of Korea; c Department of Zoology, Fakir Mohan University, Balasore, Odisha, India; d College of Pharmacy and Health Care, Tajen University, Yanpu, Taiwan; Columbia University/HHMI

**Keywords:** emerging variants, transmission pattern, mutational landscape, effect on vaccines

## Abstract

The recent emergence of multiple variants of severe acute respiratory syndrome coronavirus 2 (SARS-CoV-2) has become a significant concern for public health worldwide. New variants have been classified either as variants of concern (VOCs) or variants of interest (VOIs) by the CDC (USA) and WHO. The VOCs include lineages such as B.1.1.7 (20I/501Y.V1 variant), P.1 (20J/501Y.V3 variant), B.1.351 (20H/501Y.V2 variant), and B.1.617.2. In contrast, the VOI category includes B.1.525, B.1.526, P.2, and B.1.427/B.1.429. The WHO provided the alert for last two variants (P.2 and B.1.427/B.1.429) and labeled them for further monitoring. As per the WHO, these variants can be reclassified due to their status at a particular time. At the same time, the CDC (USA) has marked these two variants as VOIs up through today. This article analyzes the evolutionary patterns of all these emerging variants, as well as their geographical distributions and transmission patterns, including the circulating frequency, entropy diversity, and mutational event diversity throughout the genomes of all SARS-CoV-2 lineages. The transmission pattern was observed highest in the B.1.1.7 lineage. Our frequency evaluation found that this lineage achieved 100% frequency in early October 2020. We also critically evaluated the above emerging variants mutational landscape and significant spike protein mutations (E484K, K417T/N, N501Y, and D614G) impacting public health. Finally, the effectiveness of vaccines against newly SARS-CoV-2 variants was also analyzed.

## INTRODUCTION

The calamitous effects of COVID-19 are having a significant impact on existing health care systems. The pandemic has forced the world toward an economic recession. Even academic institutions have been shut down to contain the pandemic, while there has been an immense impact on communities. The industrial and the associated development sectors have plunged drastically, affecting agriculture, petroleum, manufacturing, education, information technology, research, and societal development (1). Most of the affected countries have failed to stop the pandemic. As a result, the year 2020 was the most challenging year for the world. Therefore, governments worldwide have sourced a safe and effective COVID-19 vaccine program to stop the pandemic, leading to a vaccine development race (2, 3). Global efforts by scientists led to the timely development of the COVID-19 vaccine. This was partly due to the significant efforts given by vaccine researchers as they worked around the clock to create the vaccine in 2020. At the same time, scientists from academia, pharmaceutical companies, biotechnology companies, and military organizations joined hands in developing vaccines (4), and the vaccine candidates were put into clinical trials within a span of just 6 months. The first conditional approval of the COVID-19 vaccine was given within 10 months of the COVID pandemic (5). In December 2020, the two mRNA-based COVID-19 vaccines received emergency approval, with remarkable efficacy (94 to 95%) (6, 7).

By the middle of 2020, researchers reported mutations of the severe acute respiratory syndrome coronavirus 2 (SARS-CoV-2) genome. Simultaneously several new variants came into existence and were identified through genomic data analysis. Koyama et al. reported several variants of SARS-CoV-2 in July 2020 from 10,022 genomes, obtained from four different databases. The genomes were collected and sequenced from patients from 68 countries. The study found 5,775 distinct genome variants, which included 2,969 missense mutations (8). This study affirmed that the virus was acquiring several mutations in its genome. Surprisingly, in December 2020, researchers reported a sudden rise in COVID-19 cases associated with some significant mutations, and a few of these crucial mutations were observed in the spike protein (S-protein) regions. These mutations have created several variants of SARS-CoV-2 that are more infectious among all the diversified variants (9). Reports also showed that some crucial mutations, such as D614G in some variants, might be responsible for enhanced infectivity (9, 10). Afterwards, the CDC (USA) and WHO, depending on the severity of the variants, categorized the newly emerging significant variants either as variants of concern (VOCs), variants of high consequences, or variants of interest (VOIs) (11). Three considerable VOCs that were recorded from three different regions of the world are the B.1.1.7 lineage (20I/501Y.V1 variant) from the United Kingdom, the P.1 lineage (20J/501Y.V3 variant) from Brazil, and the B.1.351 lineage (20H/501Y.V2 variant) from South Africa (12, 13). Another VOI was identified in the USA (California) and was designated B.1.427/B.1.429 (a further monitoring tag has been provided by the WHO). Consequently, a new VOC has been identified from India, which is B.1.617.2 (Delta), and this variant is now spreading worldwide. Presently, the scientific community is trying to focus on analyzing this variant and its symptomatic consequences.

Additionally, three variants are regarded as VOIs and have been identified in different parts of the world. These variants are B.1.525 (New York, USA), B.1.526 (New York, USA), and P.2 (Brazil) (Table 1). A further monitoring tag has been provided to the P.2 variant by the WHO.

**TABLE 1:** Significant SARS-CoV-2 lineages, associated variants, and their countries of origin

Serial no.	New significant SARS-CoV-2 lineage	Variant name	Variant as labeled by WHO	Country of origin	Note(s)
1	B.1.1.7	20I/501Y.V1	Alpha	United Kingdom	Emerging variant, CDC and WHO reported as VOC
2	P.1	20J/501Y.V3	Gamma	Brazil	Emerging variant, CDC and WHO declared as VOC
3	B.1.351	20H/501Y.V2	Beta	South Africa	Emerging variant, CDC and WHO stated as VOC
4	B.1.427/B.1.429	Nextstrain described as 20C/S:452R	Epsilon	USA (California)	Emerging variant, CDC and WHO reported as VOI
5	B.1.617.2	Nextstrain described as 20A/S:478K	Delta	India	Emerging variant, CDC and WHO declared as VOC
6	B.1.525	Nextstrain described as 20C	Eta	USA (New York)	CDC and WHO stated as VOI
7	B.1.526	Nextstrain described as20C	Iota	USA (New York)	CDC and WHO called as VOI
8	P.2	20J	Zeta	Brazil	CDC and WHO stated as VOI

Some crucial apprehensions have arisen regarding the variants’ infectivity, which is alarming for public health. On the other hand, another critical issue is the efficacy of the approved COVID-19 vaccines against the new variants. Some studies show that mutations in the variants may create a more infective virus. So, the question is: will such variants make a second wave or another wave of COVID-19 for different countries, creating even more new infections and triggering a more considerable death toll? Though vaccines have just been rolled out, studies have shown that variants are resistant to neutralizing antibodies, which is of considerable concern for the development of vaccines (14, 15). Furthermore, this year (2021) might be crucial due to the emergence of significant variants affecting public health and producing mutated viral antigens. These mutated antigens may affect the vaccine-generated antibodies and thus protection.

**FIG 1:**
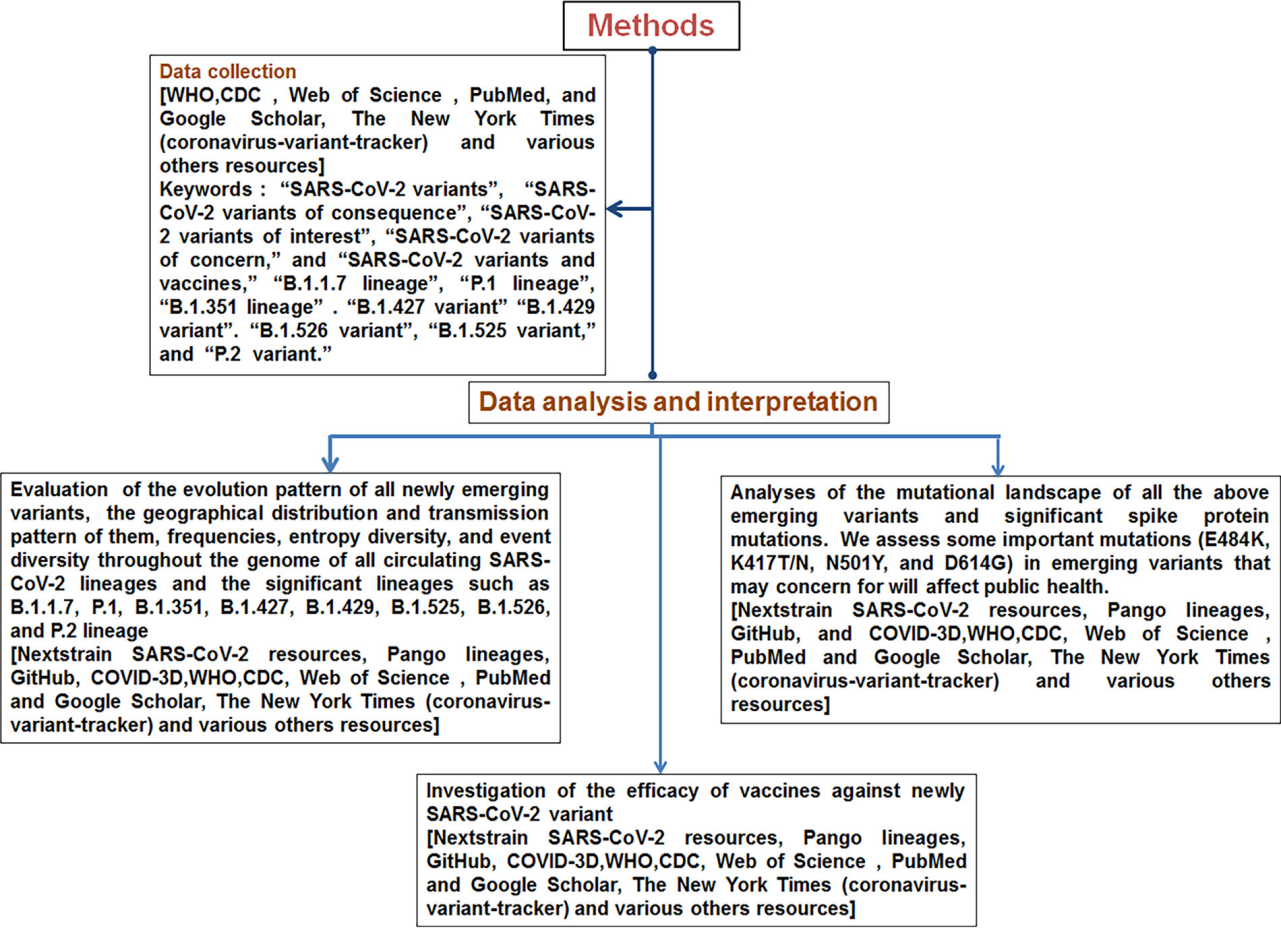
Flowchart showing the methodology of our study.

Here, we have analyzed the critical questions about the SARS-CoV-2 variants in three directions, or sections. A summary of the study methodology is provided as a flowchart in Fig. 1. In the first section, we studied the evolutionary patterns of the newly emerging variants, their geographical distributions and transmission patterns, circulating frequencies, entropy diversity, and mutational event diversity throughout the genomes of all circulating lineages of this virus and significant lineages, such as B.1.1.7, P.1, B.1.351, B.1.427/B.1.429, B.1.617.2, B.1.525, B.1.526, and P.2. In the second section, we critically evaluated the mutational landscape of all of the above emerging variants and significant spike protein mutations. We also assessed the significant mutations (E484K, K417T/N, N501Y, and D614G) in emerging variants that may concern public health. Finally, in the third section, we analyzed the efficacy of vaccines against the new SARS-CoV-2 variants. Our analysis will help design the future direction for pandemic control, next-generation vaccine development using alternative viral antigens, and planning for an effective vaccination program.

## RESULTS

### Phylogenetic tree of all circulating lineages, significant VOCs [lineages B.1.1.7 (Alpha), P.1 (Gamma), B.1.351 (Beta), and B.1.617.2 (Delta)] and significant VOIs [lineages B.1.525 (Eta), B.1.526 (Iota), P.2 (Zeta), and B.1.427/B.1.429 (Epsilon)].

**FIG 2:**
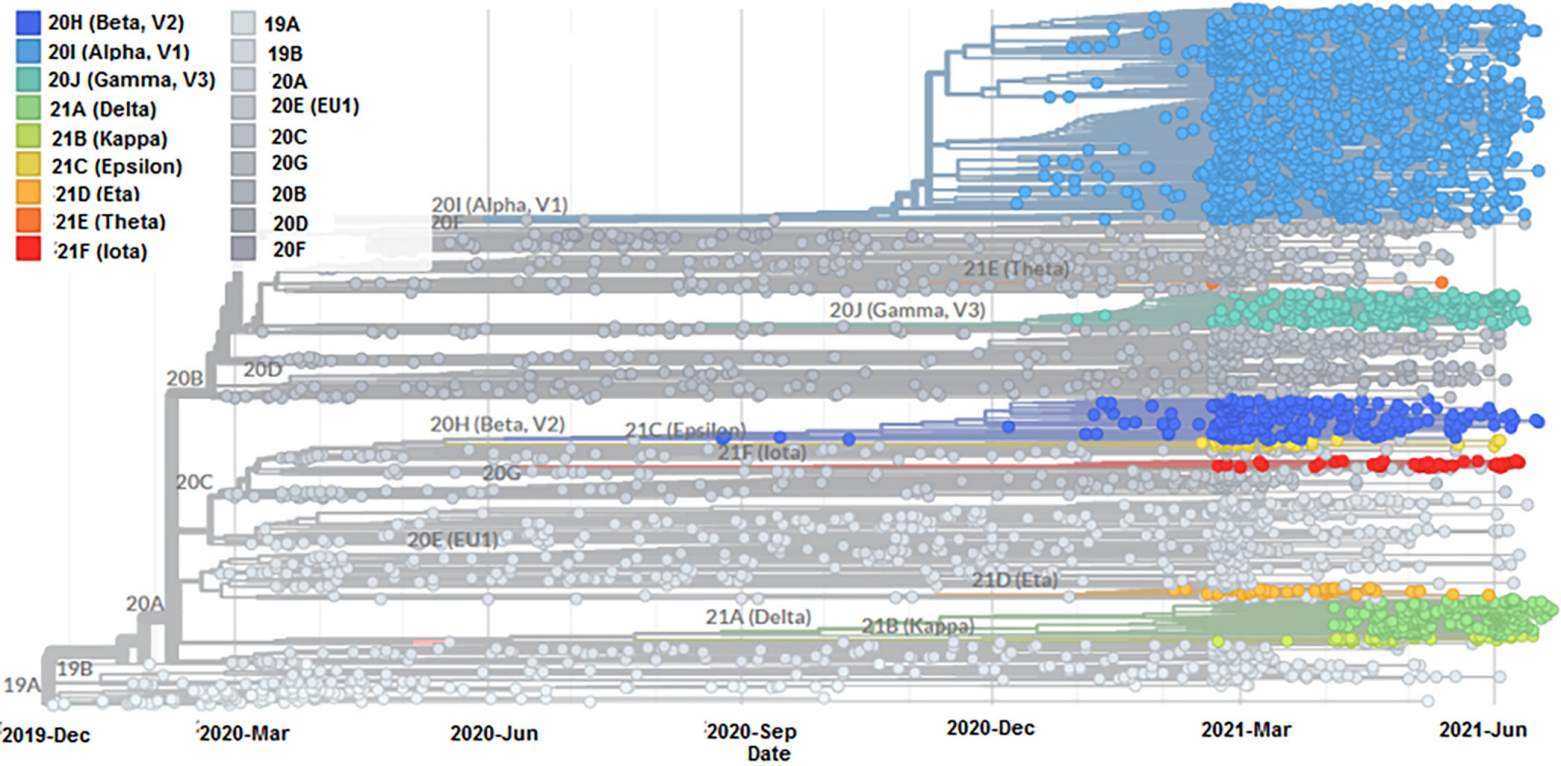
Phylogenetic tree of all circulating lineages of newly SARS-CoV-2 variants between December 2019 and June 2021. The phylogenetic tree of all circulating lineages was developed before 27 June 2021 through the Nextstrain server, using GISAID data.

**FIG 3:**
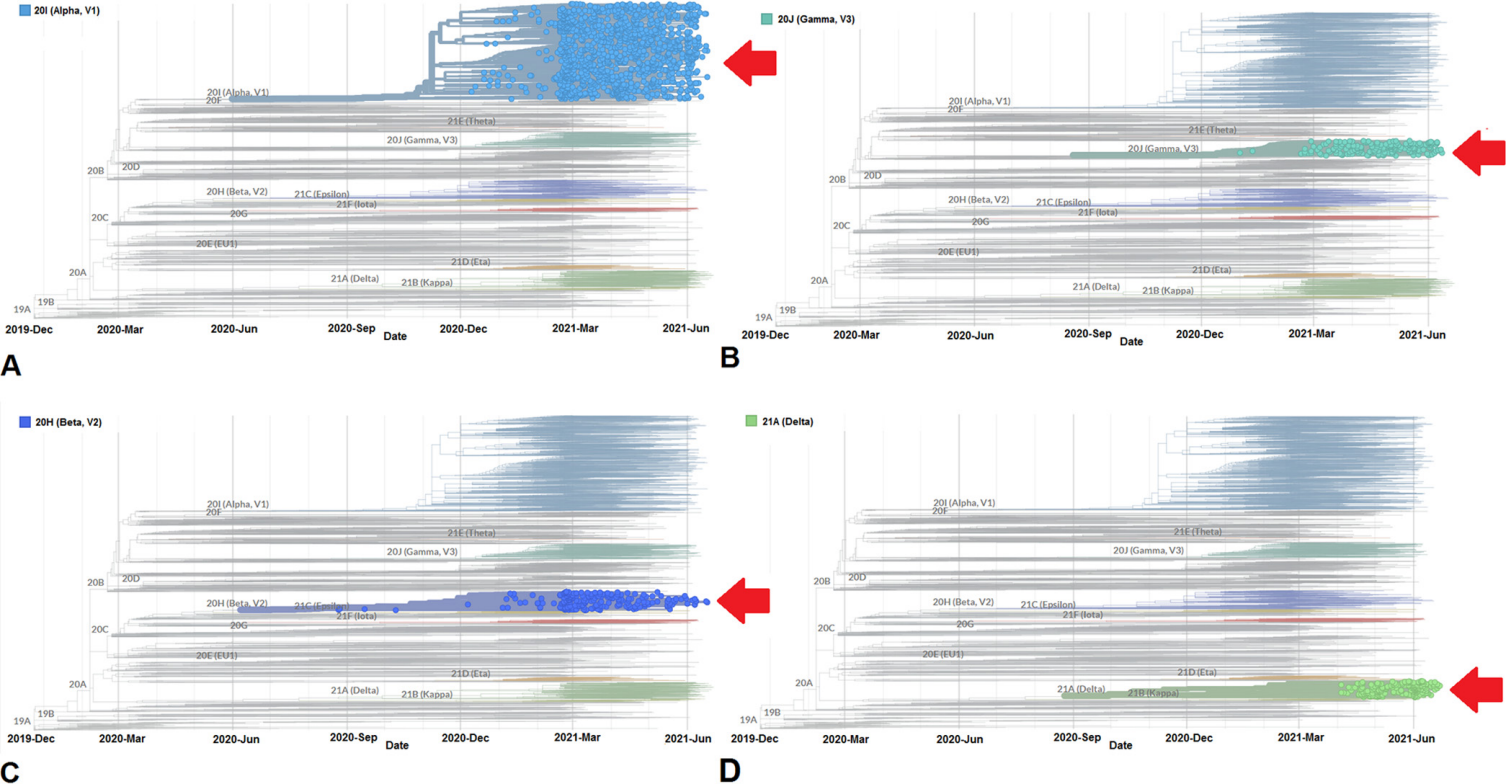
Phylogenetic trees of significant VOCs between December 2019 and June 2021. (A) Phylogenetic tree highlighting lineage B.1.1.7 (Alpha) and showing the relationship with other circulating lineages. (B) Phylogenetic tree highlighting lineage P.1 (Gamma) and showing the relationship with different circulating lineages. (C) Phylogenetic tree highlighting lineage B.1.351 (Beta) and showing the relationship with other circulating lineages. (D) Phylogenetic tree showing lineage B.1.617.2 (Delta) and showing the relationship with different circulating lineages. The phylogenetic trees of significant VOCs were developed before 27 June 2021 through the Nextstrain server, using GISAID data.

We have developed a phylogenetic tree for all the circulating SARS-CoV-2 lineages between December 2019 and June 2021, and for this, 3,905 genomes were sampled (Fig. 2). We have analyzed molecular phylogenetics and developed a tree for lineage B.1.1.7. We found 1,182 genome samples for lineage B.1.1.7 (20I/501Y.V1) between December 2020 and June 2021 from a total of 3,905 genomes (Fig. 3A). The data from the development of the phylogenetic tree depict several significant mutations acquired by the lineage B.1.1.7 since December 2020.

Similarly, we have developed a phylogenetic tree of lineage P.1 and found 186 genome samples of lineage P.1 (20J/501Y.V3 variant) between December 2020 and June 2021 from the total of 3,905 genomes (Fig. 3B). The phylogenetic tree reflects that some significant mutations had been acquired by lineage P.1 (20J/501Y.V3 variant) since the middle of December 2020.

Furthermore, we developed a phylogenetic tree of lineage B.1.351 and found 231 genome samples for lineage B.1.351 (20H/501Y.V2) between December 2020 and June 2021 from the total of 3,905 genomes (Fig. 3C). Both the phylogenetic tree and the timeline indicate that lineage B.1.351 has acquired some significant mutations since September 2020.

Another phylogenetic tree of lineage B.1.617.2 (Delta) found 201 genome samples of lineage B.1.617.2 between December 2020 and June 2021 from 3,905 genomes (Fig. 3D). The lineage B.1.617.2 was first identified in India in late 2020. The phylogenetic tree indicates that a small number of significant mutations were acquired by lineage B.1.617.2 since the middle of March 2021.

**FIG 4:**
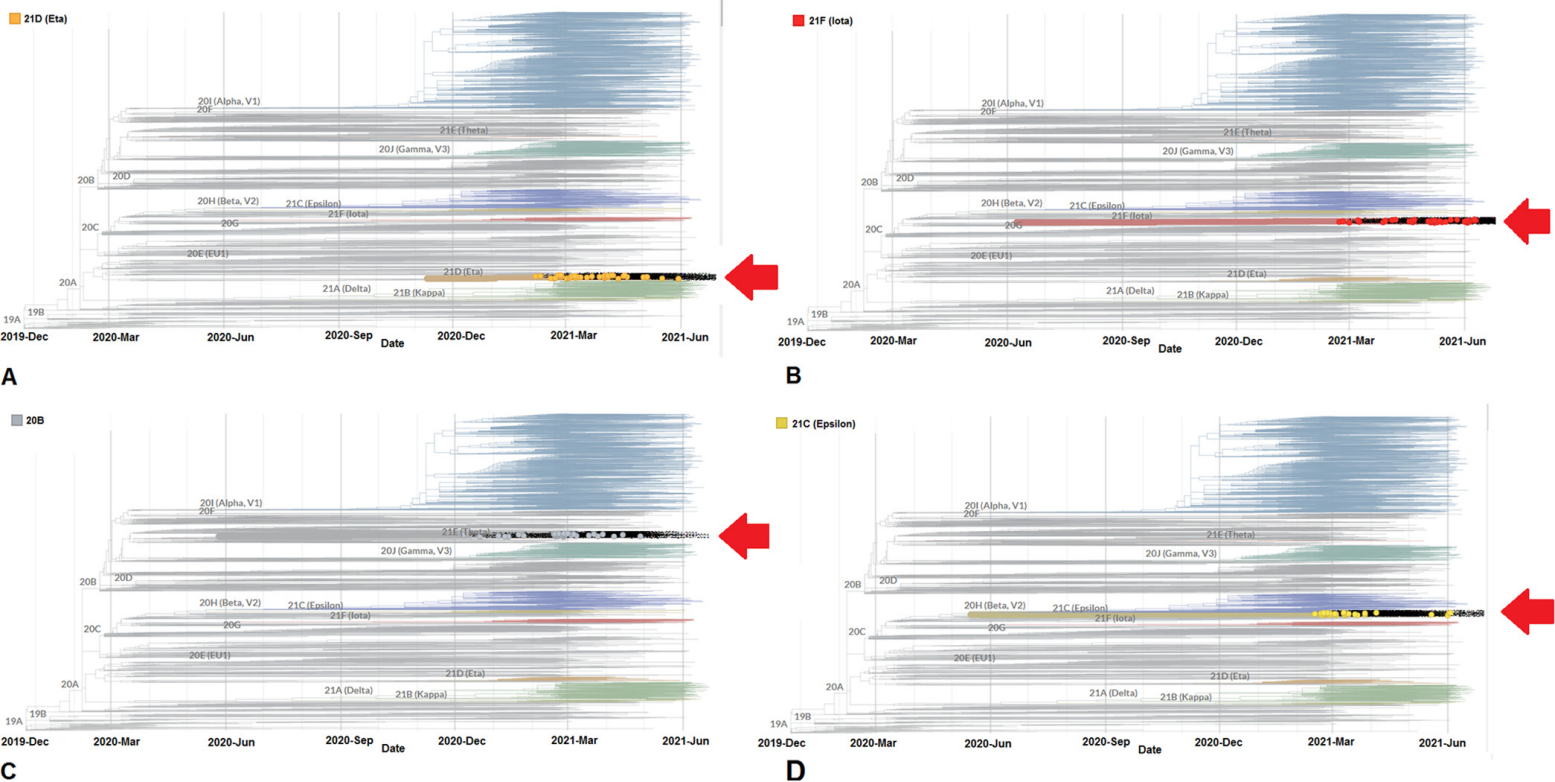
Phylogenetic trees of significant VOIs between December 2019 and June 2021. (A) Phylogenetic tree highlighting lineage B.1.525 (Eta) and showing the relationship with other circulating lineages. (B) Phylogenetic tree highlighting lineage B.1.526 (Iota) and showing the relationship with different circulating lineages. (C) Phylogenetic tree highlighting lineage P.2 (Zeta) and showing the relationship with other circulating lineages. (D) Phylogenetic tree highlighting lineage B.1.427/B.1.429 (Epsilon) and showing the relationship with other circulating lineages. The phylogenetic trees of significant VOIs were developed before 27 June 2021 through the Nextstrain server, using GISAID data.

In addition, we have developed a phylogenetic tree of lineage B.1.525 and found 45 genome samples of the B.1.525 lineage between December 2020 and June 2021 from the total of 3,905 genomes (Fig. 4A). The B.1.425 lineage was found in the USA. The phylogenetic tree indicates that a few mutations have been acquired by lineage B.1.525 since February 2021. It was observed that all the samples of this lineage have formed a cluster since February 2021.

The phylogenetic tree of lineage B.1.526 found 41 genome samples of lineage B.1.526 between December 2020 and June 2021 from the 3,905 genomes (Fig. 4B). Lineage B.1.426 was also found in the USA. The phylogenetic tree shows that many mutations have been gained by lineage B.1.526 since the middle of February 2021.

Likewise, we depict the phylogenetic tree of lineage P.2, and 41 genome samples of the P.2 lineage between December 2020 and June 2021 from the 3,905 genomes were observed (Fig. 4C). It appears from the phylogenetic tree that some important mutations have been obtained by the P.2 lineage since the middle of December 2020.

Next, we also created a phylogenetic tree of lineage B.1.427/B.1.429 and found 35 genome samples for lineage B.1.427/B.1.429 (Epsilon) between December 2020 and June 2021 from 3,905 genomes (Fig. 4D). This lineage was found in the USA. The phylogenetic tree indicates there have been few significant mutations acquired by this lineage since February 2021.

### Scatterplot of cluster analysis of all circulating lineages, significant VOCs [lineages B.1.1.7 (Alpha), P.1 (Gamma), B.1.351 (Beta), and B.1.617.2 (Delta)], and significant VOIs [lineages B.1.525 (Eta), B.1.526 (Iota), P.2 (Zeta), and B.1.427/B.1.429 (Epsilon)].

**FIG 5:**
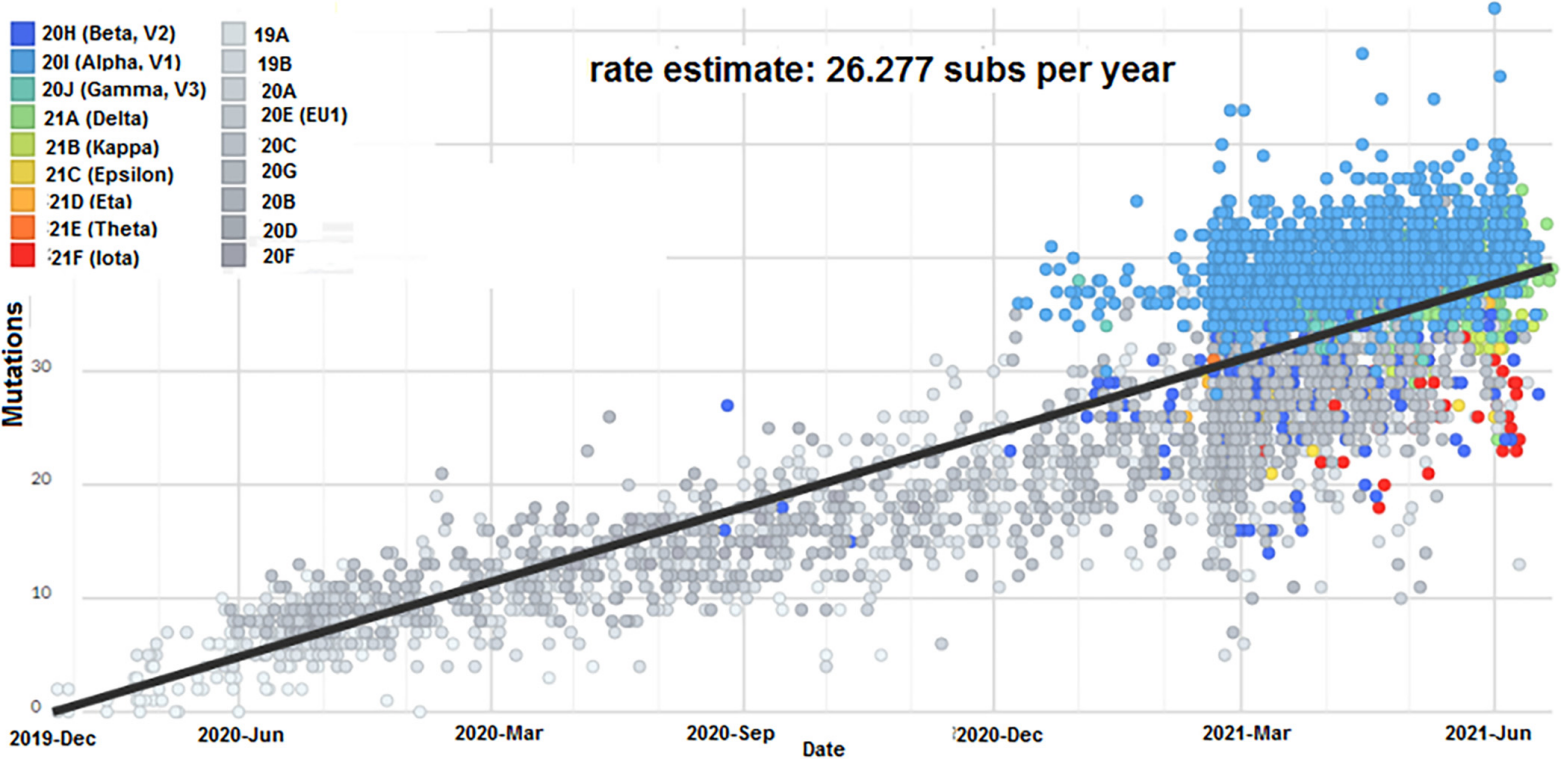
Scatterplot showing the genome diversity cluster of all circulating lineages between December 2019 and June 2021. The scatterplot of all circulating lineages was developed before 27 June 2021 through the Nextstrain server, using GISAID data.

**FIG 6:**
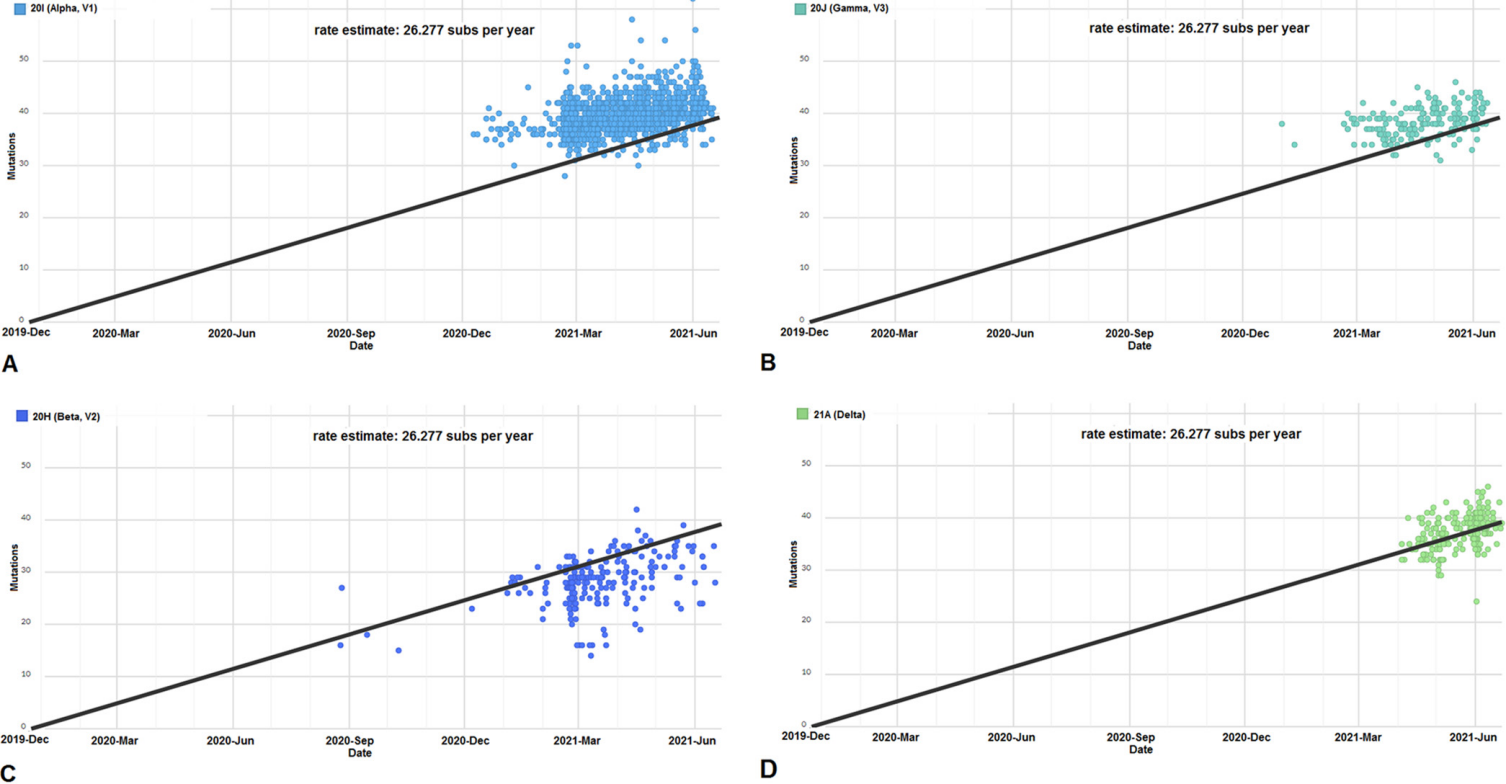
Scatterplots showing the genome diversity clusters of significant VOCs between December 2019 and June 2021. (A) Scatterplot showing the genome samples of lineage B.1.1.7 (Alpha) with a linear regression line. (B) Scatterplot showing the genome samples of lineage P.1 (Gamma) with a linear regression line. (C) Scatterplot showing the genome samples of lineage B.1.351 (Beta) with a linear regression line (D) Scatterplot showing the genome samples of lineage B.1.617.2 (Delta) with a linear regression line. The scatterplots of significant VOCs were developed before 27 June 2021 through the Nextstrain server, which is using GISAID data.

We performed a cluster analysis and developed scatterplots to show the clustering of the genome samples and the divergence in mutations along the regression line. Scatterplots with a linear regression line using 3,905 genomes of all circulating lineages were developed (Fig. 5). Our linear regression model shows that the sample values are distributed just alongside the regression line. The scatters in the plot describe the overall pattern of all circulating lineages through the plotted points. The pattern of the linear regression model demonstrates that this regression model follows a dense distribution pattern of samples on both sides of the line. The model also portrays that the genome samples on the upper side are more viscous than the lower ones. This regression model shows a heteroscedasticity pattern. We next developed scatterplots using 1,182 genomes of the B.1.1.7 lineage (Fig. 6A). The B.1.1.7 lineage’s scatterplot describes the B.1.17 lineage pattern through the plotted points. In the regression model of the B.1.1.7 lineage, the sample points are densely plotted on the upper side of the top portion of the regression line. Simultaneously, we also developed scatterplots using 186 genomes of the P.1 lineage (Fig. 6B). The regression model describes the pattern of the P.1 lineage through the plotted points. The scatterplot pattern of the P.1 lineage shows that the samples form a cluster on the upper side of the top portion of the regression line. This regression model is quite similar to the pattern of the B.1.1.7 lineage regression model. Next, we developed scatterplots using 231 genomes of the B.1.351 lineage (Fig. 6C). The scatterplot of the B.1.351 lineage illustrates the pattern through the plotted points. The scatterplot pattern represents that the samples form a cluster mainly on the lower side of the top portion of the regression line. This regression model shows that the data points of this cluster are dense in the upper portion. From this model, it can be inferred that most of the mutations were accumulated from March 2021 to June 2021. Likewise, a scatterplot using 201 genomes of the B.1.617.2 lineage was also plotted (Fig. 6D). The scatterplot trend demonstrated that the samples form a cluster on both the sides of the top portion of the regression line. From this regression model, it can be inferred that the Delta variant accumulated maximum mutations from April/May 2021 to June 2021.

**FIG 7:**
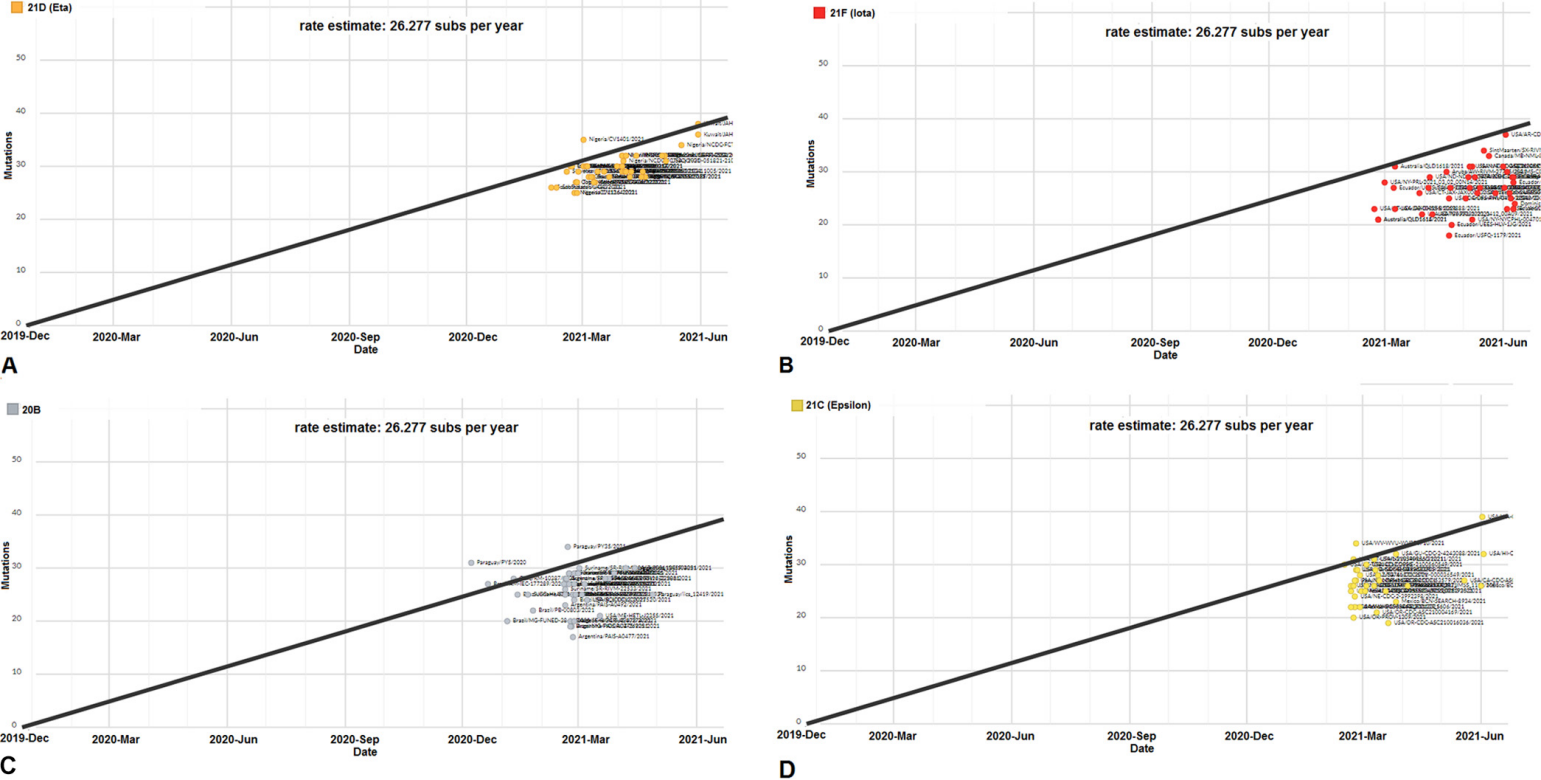
Scatterplots showing the genome diversity cluster of significant VOIs between December 2019 and June 2021. (A) Scatterplot showing the genome samples of the lineage B.1.525 (Eta) with a linear regression line. (B) Scatterplot showing the genome samples of the lineage B.1.526 (Iota) with a linear regression line. (C) Scatterplot showing the genome samples of the lineage P.2 (Zeta) with a linear regression line. (D) Scatterplot showing the genome samples of the lineage B.1.427/B.1.429 (Epsilon) with a linear regression line. The scatterplots of significant VOIs were developed before 27 June 2021 through the Nextstrain server, using GISAID data.

A scatterplot using 45 genomes of the B.1.525 lineage (Fig. 7A) was plotted, and it confirmed that the B.1.425 lineage forms a cluster on the lower side of the regression line's top portion. This regression model informed us that the variant accumulated maximum mutations from February/March 2021 to June 2021. We next developed scatterplots using 41 genomes of the B.1.526 lineage (Fig. 7B). It demonstrated that the B.1.526 lineage forms a cluster mostly at the lower side of the top portion of the regression line. This regression model follows the same pattern as the B.1.425 lineage and shows that the variant accumulated maximum mutations from March 2021 to June 2021. Next, we developed a scatterplot using 41 genomes of the P.2 lineage (Fig. 7C). The scatterplot trend established that the P.2 lineage forms a cluster mostly at the lower side of the top portion of the regression line. According to this regression model, the P.2 lineage accumulated more mutations from December 2020 to May 2021 and maximum mutations during March 2021. Next a scatterplot using 35 genomes of the B.1.427/B.1.429 lineage was plotted (Fig. 7D). The scatterplot trend demonstrated that the samples form a cluster on the lower side of the top portion of the regression line. This regression model shows the same pattern as the previous regression model of the B.1.351 lineage. This model also informs us that the variant accumulated more mutations from March 2021 to June 2021. From the scatterplot and the regression model, we can observe that all VOIs show the same type of scattered plot.

### Geographical distributions and transmission patterns from origin to other countries of all circulating lineages, significant VOCs [lineages B.1.1.7 (Alpha), P.1 (Gamma), B.1.351 (Beta), and B.1.617.2 (Delta)], and significant VOIs [lineages B.1.525 (Eta), B.1.526 (Iota), P.2 (Zeta), and B.1.427/B.1.429 (Epsilon)].

**FIG 8:**
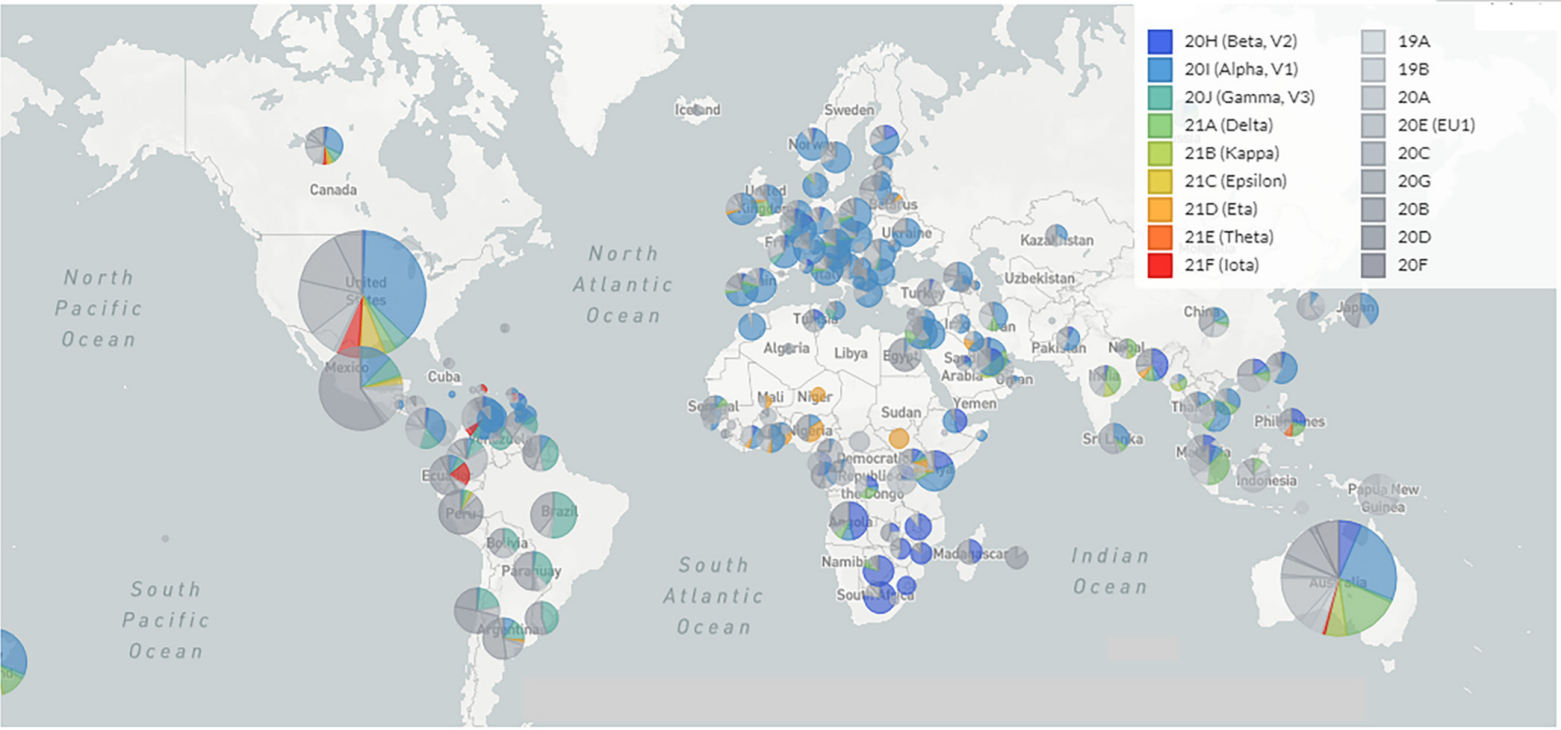
Geographical distribution and transmission pattern from the source to other countries of all circulating lineages between December 2019 and June 2021. The geographical distribution and transmission pattern of all circulating lineages were developed before 27 June 2021 through the Nextstrain server, using GISAID data.

**FIG 9:**
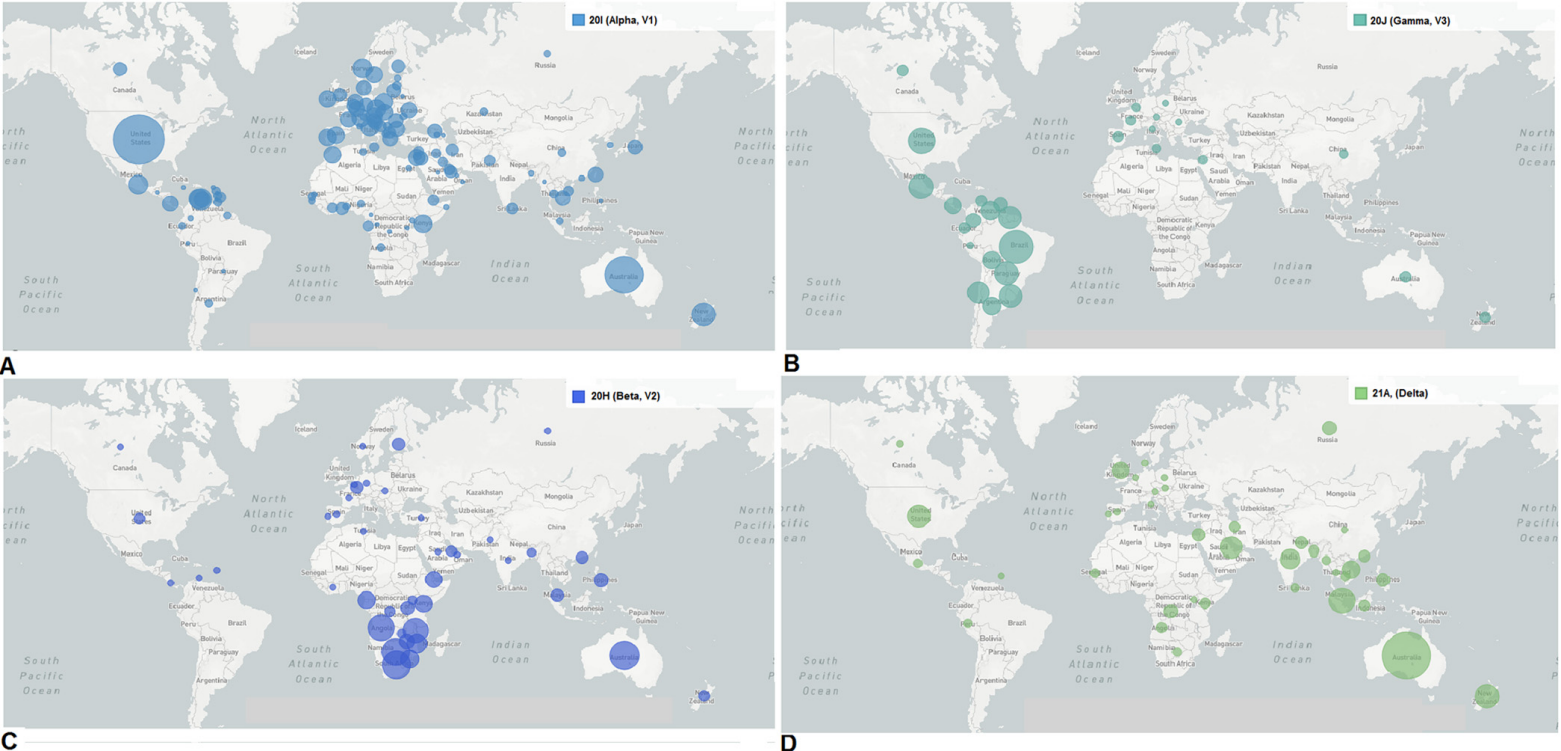
Geographical distributions and transmission patterns from the source to other countries of significant VOCs between December 2019 and June 2021. (A) Geographical distribution and transmission pattern of the B.1.1.7 (Alpha) lineage. (B) Geographical distribution and transmission pattern of the P.1 (Gamma) lineage. (C) Geographical distribution and transmission pattern of the B.1.351 (Beta) lineage. (D) Geographical distribution and transmission pattern of the B.1.617.2 (Delta) lineage. The geographical distributions and transmission patterns of all circulating lineages were developed before 27 June 2021 through the Nextstrain server, using GISAID data.

We have mapped the geographical distributions and transmission patterns of all circulating lineages at one time (Fig. 8). Genomic diversity is prominent in different regions of the world. We analyzed the geographical distribution and transmission of B.1.1.7 lineage and depict them in Fig. 9A. This lineage originated from the United Kingdom. It is very clear from the transmission pattern that the lineage got transmitted throughout Europe, the USA, and Canada, including parts of Latin America and South Africa. The geographical distribution and transmission of the P.1 lineage are recorded in Fig. 9B. The lineage originated in Brazil and got transmitted to different parts of South America. The lineage was also reported in France. The geographical distribution and transmission of B.1.351 lineage are shown in Fig. 9C. The lineage originated in South Africa and then got transmitted to different parts of Europe, Asia (India, Sri Lanka, and Malaysia), and Australia.

Similarly, the geographical distribution and transmission of the B.1.617.2 lineage are recorded in Fig. 9D. This variant originated in the USA and then got transmitted to different parts of the United Kingdom and USA.

**FIG 10:**
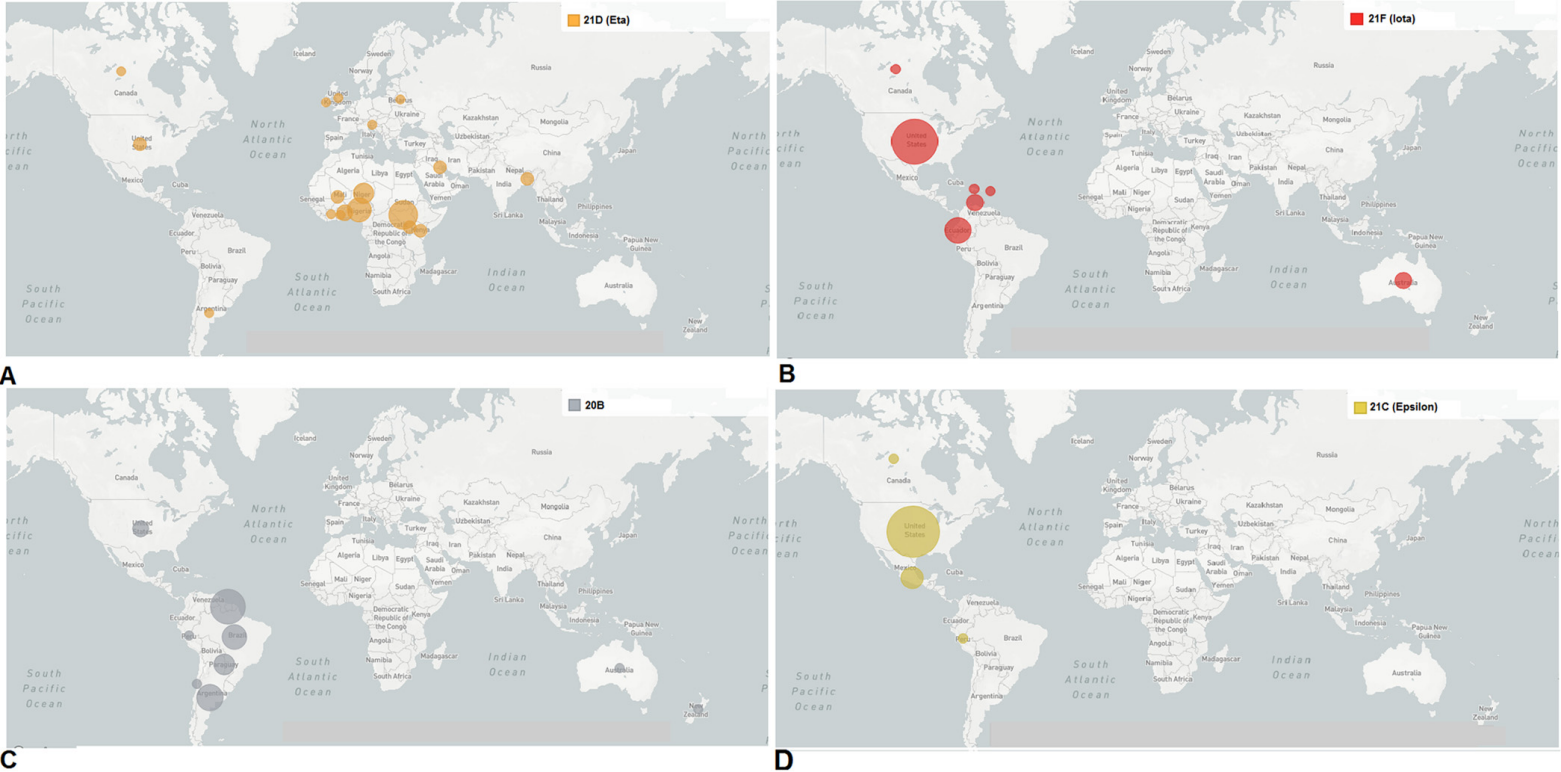
Geographical distributions and transmission patterns from the source to other countries of significant VOIs between December 2019 and June 2021. (A) Geographical distribution and transmission pattern of the B.1.525 (Eta) lineage. (B) Geographical distribution and transmission pattern of the B.1.526 (Iota) lineage. (C) Geographical distribution and transmission pattern of the P.2 (Zeta) lineage. (D) Geographical distribution and transmission pattern of the B.1.427/B.1.429 (Epsilon) lineage. The geographical distributions and transmission patterns of all circulating lineages were developed before 27 June 2021 through the Nextstrain server, using GISAID data.

The geographical distribution and transmission of the B.1.525 lineage are shown in Fig. 10A. This variant originated in the USA and got transmitted to different parts of South Africa and Australia.

We mapped the geographical distributions and transmission of the B.1.526 lineage and recorded them in Fig. 10B. The B.1.526 lineage originated in the USA and later got transmitted to different parts of South America. In the end, we have drawn the geographical distribution and transmission of the P.2 lineage (Fig. 10C). The lineage originated in Brazil and then got transmitted to different areas of South America, parts of the USA, and Canada.

The geographical distribution and transmission of lineage B.1.427/B.1.429 are noted in Fig. 10D. It originated in the USA and then got transmitted to different parts of Latin America, including Argentina.

### Circulating frequencies of all SARS-CoV-2 lineages, significant VOCs (lineages B.1.1.7 (Alpha), P.1 (Gamma), B.1.351 (Beta), and B.1.617.2 (Delta)], and significant VOIs (lineages B.1.525 (Eta), B.1.526 (Iota), P.2 (Zeta), and B.1.427/B.1.429 (Epsilon)] over time.

**FIG 11:**
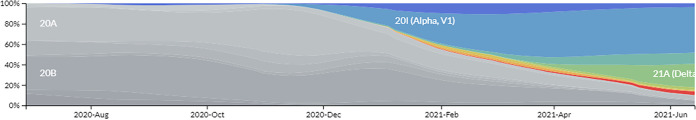
Frequency pattern of all circulating lineages between December 2019 and June 2021. The frequency pattern of all circulating lineages was developed before 27 June 2021 through the Nextstrain server, using GISAID data.

**FIG 12:**
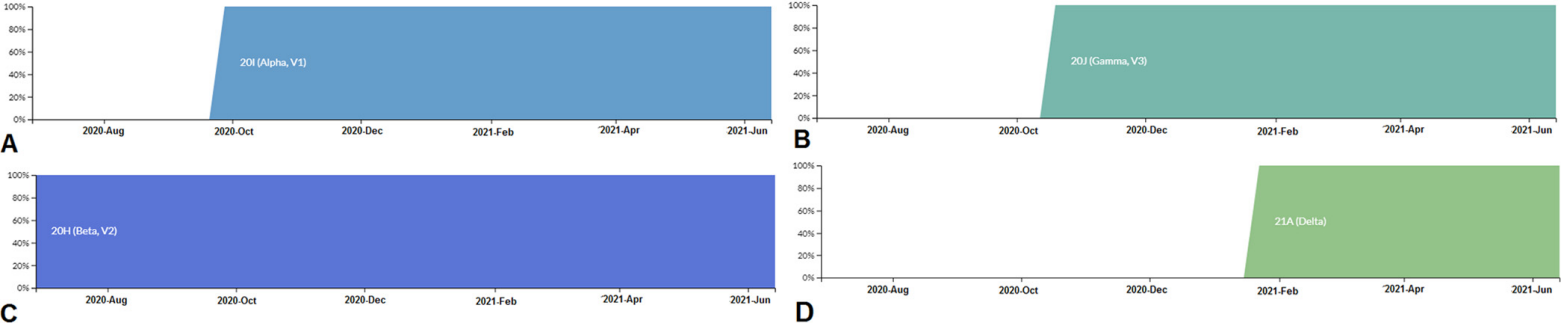
Frequency patterns of significant VOCs between December 2019 and June 2021. (A) Frequency pattern of the B.1.1.7 (Alpha) lineage. (B) Frequency pattern of the P.1 (Gamma) lineage. (C) Frequency pattern of the B.1.351 (Beta) lineage. (D) Frequency pattern of the B.1.617.2 (Delta) lineage. The frequency patterns of significant VOCs were developed before 27 June 2021 through the Nextstrain server, using GISAID data.

We mapped the circulating frequencies of all SARS-CoV-2 lineages in a framework and recorded them in Fig. 11. The circulating frequency of the B.1.1.7 lineage is noted in Fig. 12A. The circulating frequency indicated that it originated in September, and it achieved 100% frequency in early October 2020. We next analyzed the circulating frequency of the P.1 lineage and recorded it in Fig. 12B. The data indicated that this variant achieved 100% frequency in the middle of October 2020. The circulating frequency of the B.1.351 lineage is represented in Fig. 12C. It was observed that the lineage achieved 100% frequency between June and July 2020.

Similarly, we evaluated the circulating frequencies of the B.1.617.2 lineage, as shown in Fig. 12D. It was noted that the lineage achieved 100% frequency during the last week of January 2021 and early February 2021.

**FIG 13:**
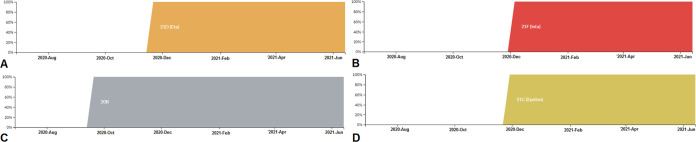
Frequency patterns of significant VOIs between December 2019 and June 2021. (A) Frequency pattern of the B.1.525 (Eta) lineage. (B) Frequency pattern of the B.1.526 (Iota) lineage. (C) Frequency pattern of the P.2 (Zeta) lineage. (D) Frequency pattern of the B.1.427/B.1.429 (Epsilon) lineage. The frequency patterns of significant VOIs were developed before 27 June 2021 through the Nextstrain server, using GISAID data.

Furthermore, the circulating frequency of B.1.525 lineage is noted in Fig. 13A. It achieved 100% frequency during November 2020. At the same time, we mapped the circulating frequency of the B.1.526 lineage (Fig. 13B). This variant achieved 100% frequency during the first week of December 2020. Finally, we have drawn the circulating frequency pattern of the P.2 lineage (Fig. 13C). The frequency was recorded as 100% at the end of September 2020 or early October 2020. The circulating frequency of lineage B.1.427/B.1.429 is noted in Fig. 13D. The lineage achieved 100% frequency during early December 2020.

### Entropy diversity and mutational event diversity throughout the genomes of all circulating lineages, significant VOCs [lineages B.1.1.7 (Alpha), P.1 (Gamma), B.1.351 (Beta), and B.1.617.2 (Delta)], and significant VOIs [lineages B.1.525 (Eta), B.1.526 (Iota), P.2 (Zeta), and B.1.427/B.1.429 (Epsilon)].

**FIG 14:**
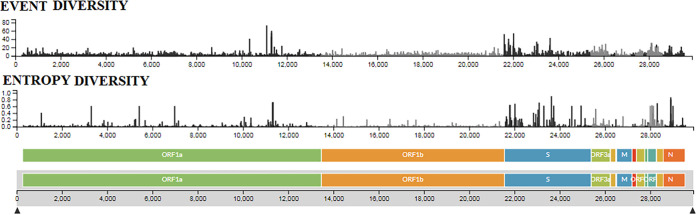
Entropy diversity and event diversity of all circulating lineages between December 2019 and June 2021. The entropy diversity and event diversity of all circulating lineages were developed before 27 June 2021 through the Nextstrain server, using GISAID data.

**FIG 15:**
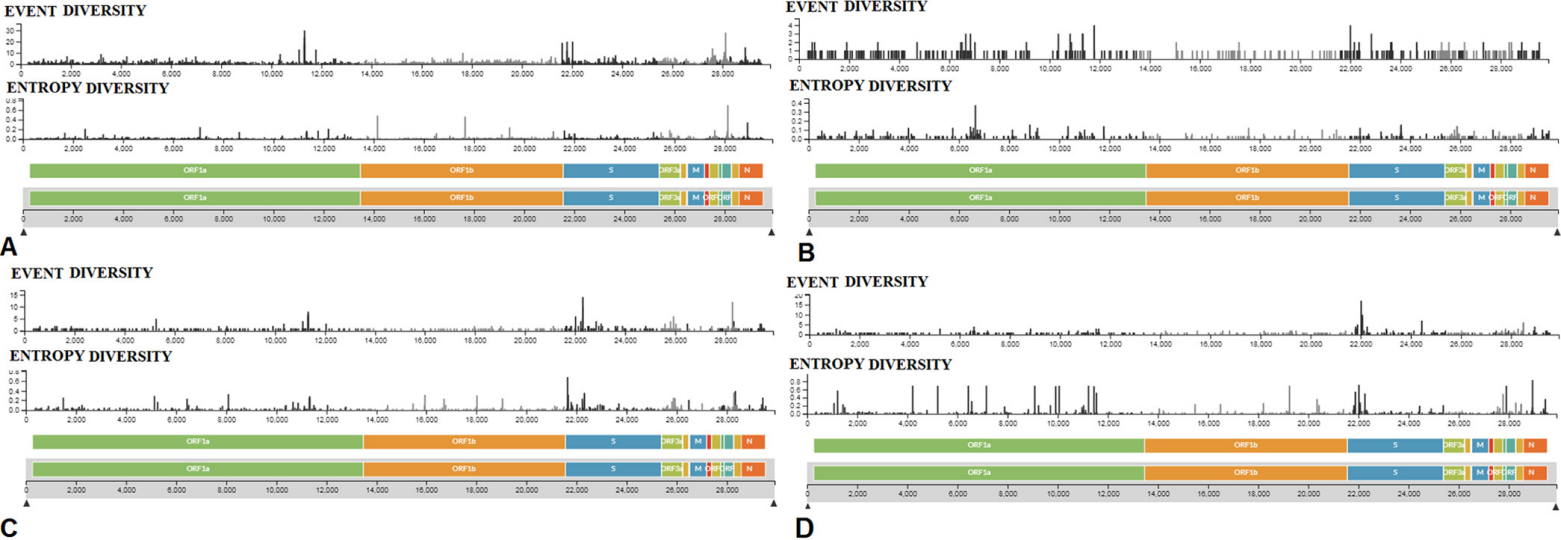
Entropy diversities and event diversities of significant VOCs between December 2019 and June 2021. (A) Entropy diversity and event diversity of the B.1.1.7 (Alpha) lineage. (B) Entropy diversity and event diversity of the P.1 (Gamma) lineage. (C) Entropy diversity and event diversity of the B.1.351 (Beta) lineage. (D) Entropy diversity and event diversity of the B.1.617.2 (Delta) lineage. Entropy diversity and event diversity of significant VOCs were developed before 27 June 2021 through the Nextstrain server, using GISAID data.

Entropy diversity is a measure to understand the pattern of mutational changes in a particular position in the genome. At the same time, it also helps us to understand the tendency of amino acids to swap from wild type to mutant (16). Similarly, mutational event diversity or mutational profiling studies inform us about the mutational events throughout the genome or at a specific position. Such studies can assist us in understanding the mechanisms that cause the SARS-CoV-2 evolution (17). Thus, we have depicted the entropy diversity and mutational event pattern throughout the genomes of all circulating lineages in a frame (Fig. 14). Based on the entropy pattern and mutational event points throughout the genome of the B.1.1.7 lineage (Fig. 15A), there was a maximum entropy of 0.8, and in two positions in ORF1b, the entropy was noted nearby as 0.6. The entropy diversity and event diversity points throughout the genome of P.1 lineage are depicted in Fig. 15B. In this case, maximum entropy was noted as 0.4, but it was pointed out in a position in ORF1a. The entropy diversity and mutational event plots throughout the genome of the B.1.351 lineage are depicted in Fig. 15C. Maximum entropy in this case was noted between 0.6 and 0.8, but it was pointed out in a position in S-protein. Similarly, we evaluated the entropy diversity and mutational event plot throughout the genome of the B.1.617.2 lineage (Fig. 15D). In this case, also, maximum entropy was noted at about 0.8, but it was noted at different positions (nine positions in ORF1a, one position in ORF1b, one position in S-protein, and one position in N-protein).

**FIG 16:**
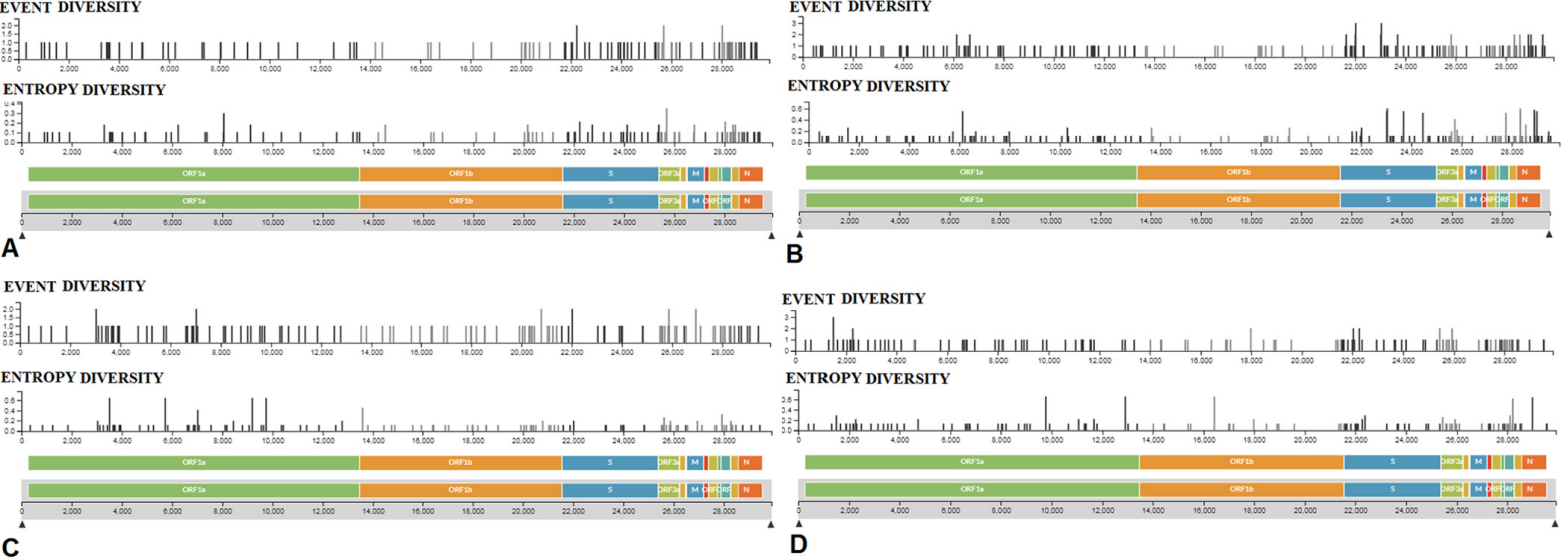
Entropy diversity and event diversity of significant VOIs between December 2019 and June 2021. (A) Entropy diversity and event diversity of the B.1.525 (Eta) lineage. (B) Entropy diversity and event diversity of the B.1.526 (Iota) lineage. (C) Frequency pattern of the P.2 (Zeta) lineage. (D) Entropy diversity and event diversity of the B.1.427/B.1.429 (Epsilon) lineage. The entropy diversities and event diversities of significant VOIs were developed before 27 June 2021 through the Nextstrain server, using GISAID data.

Again, we have depicted the entropy diversity and mutational event pattern plot throughout the genome of the B.1.525 lineage (Fig. 16A). The maximum entropy was noted at about 0.4 in ORF3a. At two positions in ORF1a, the entropy observed was about 0.3.

At the same time, we mapped the entropy diversity and mutational events throughout the genome of the B.1.526 lineage (Fig. 16B). In this case, the maximum entropy noted was about 0.6 in different positions.

Furthermore, we have drawn the entropy diversity and mutational event pattern throughout the genome of the P.2 lineage (Fig. 16C). In this case, the maximum entropy noted was about 0.6 in four different positions in ORF1a. Finally, we recorded the entropy diversity and mutational event plots throughout the genome of lineage B.1.427/B.1.429 (Fig. 16D). Here, the maximum entropy noted was about 0.6 in five different positions (two positions in ORF1a, one position in ORF1b, and one position in N-protein).

### Viral mutational landscapes of all major VOCs and VOIs, their significant mutations, and significant mutations in spike protein.

**FIG 17:**
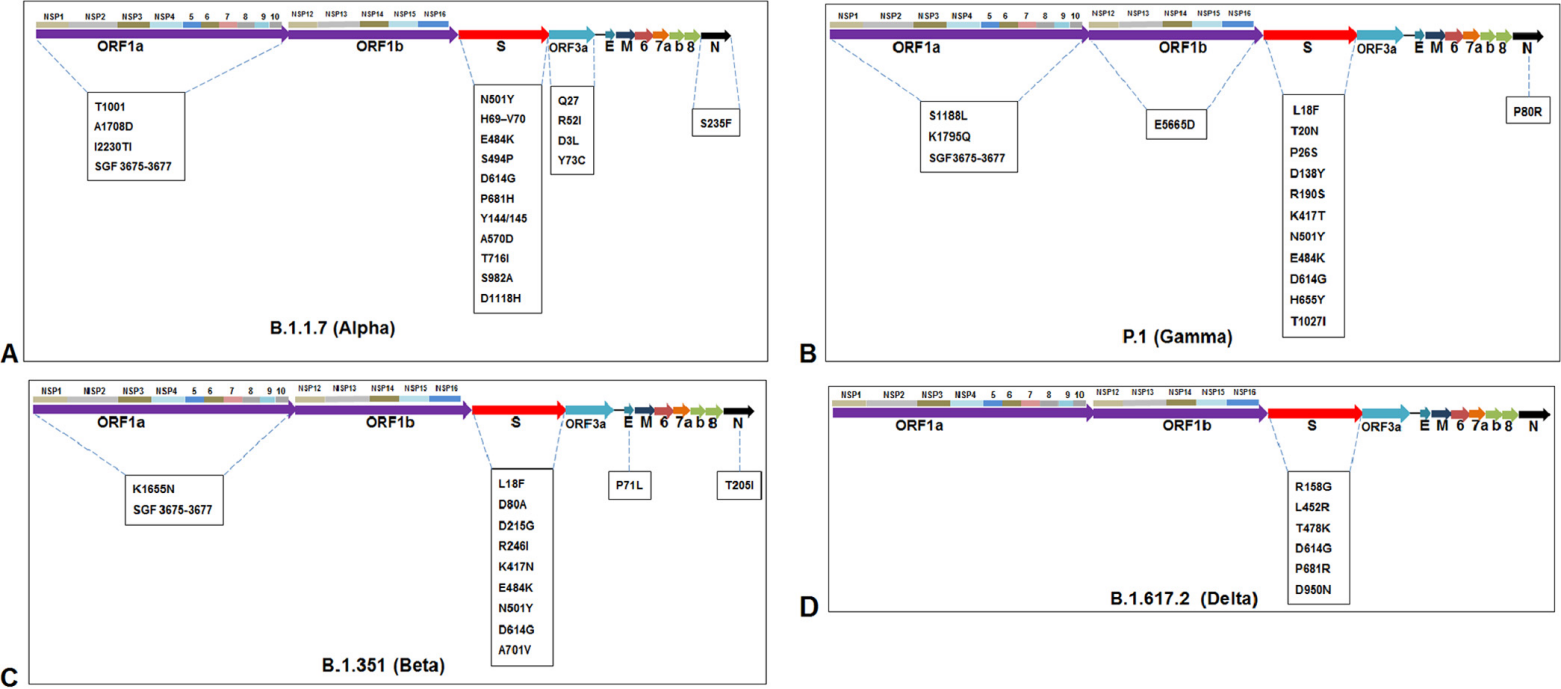
Mutational landscapes throughout the genomes of significant VOCs between December 2019 and June 2021. (A) Significant mutational landscape throughout the genome of the B.1.1.7 (Alpha) lineage. (B) Significant mutational landscape throughout the genome of the P.1 (Gamma) lineage. (C) Significant mutational landscape throughout the genome of the B.1.351 (Beta) lineage. (D) Significant mutational landscape throughout the genome of the B.1.617.2 (Delta) lineage.

**FIG 18:**
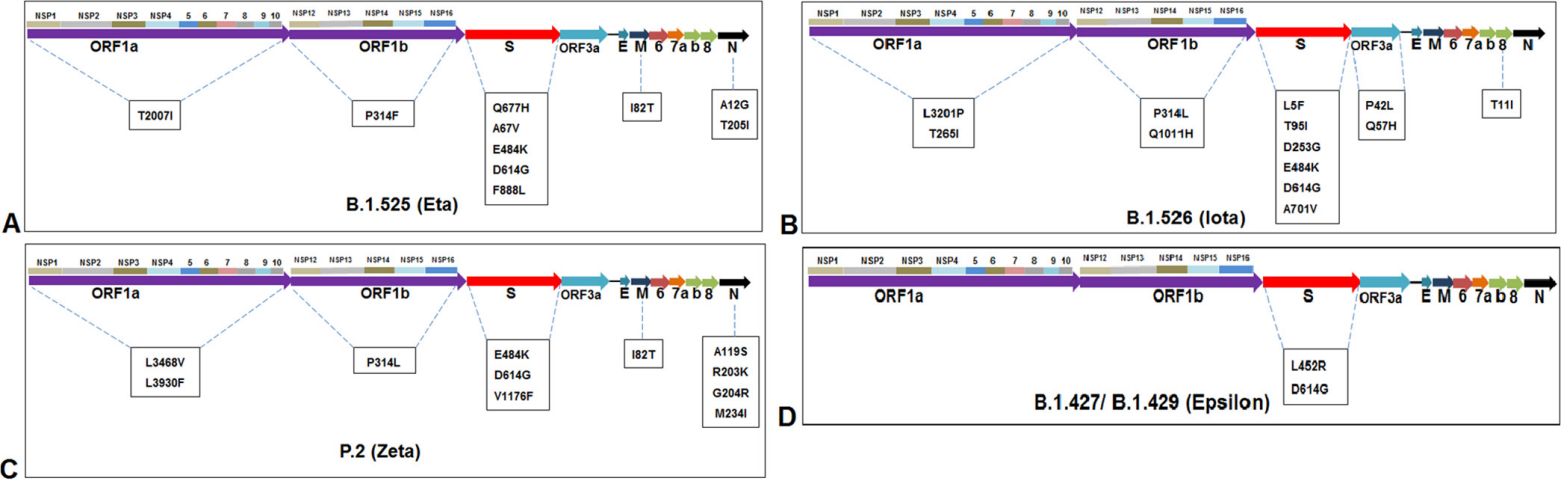
Mutational landscapes throughout the genomes of significant VOIs between December 2019 and June 2021. (A) Significant mutational landscape throughout the genome of the B.1.525 (Eta) lineage. (B) Significant mutational landscape throughout the genome of the B.1.526 (Iota) lineage. (C) Significant mutational landscape throughout the genome of the P.2 (Zeta) lineage. (D) Significant mutational landscape throughout the genome of the B.1.427/B.1.429 (Epsilon) lineage.

The mutational landscapes of all emerging variants (VOC and VOI), as reported by the CDC and WHO, are shown in Tables 2 and 3. The mutational landscapes and the significant mutational positions are described through the schematic illustration for all major VOCs, such as lineages B.1.1.7 (Fig. 17A), P.1 (Fig. 17B), B.1.351 (Fig. 17C), and B.1.617.2 (Fig. 17D). At the same time, the mutational landscapes and the significant mutational positions are described through the schematic illustration for all major VOIs, such as lineages B.1.525 (Fig. 18A), B.1.526 (Fig. 18B), P.2 (Fig. 18C), and B.1.427/B.1.429 (Fig. 18D).

**TABLE 2:** Mutational landscape of different emerging variants of concern

Variant name	Mutation	Mutation position	Amino acid change
B.1.1.7	N501Y	Receptor binding domain of spike protein	Asn^501^→Tyr
	H69–V70	Spike protein	Deletion mutation
	P681H	S1/S2 furin cleavage site of spike protein	Pro^681^→His
	Y144/145	Spike deletion	Deletion mutation
	A570D	Spike glycoprotein subunit1	Ala^570^→Asp
	T716I	Spike glycoprotein chain A	Thr^7161^→Ile
	S982A	Spike glycoprotein chain A	Ser^982^→Ala
	D1118H	Spike glycoprotein chain A	Asp^1118^→His
	Q27	ORF3a	Stop codon generated due to mutation in 27^th^ position
	T1001I	ORF1a	Thr^1001^→Ile
	A1708D	ORF1a	Ala^1708^→Asp
	I2230T	ORF1a	Ile^12230^→Thr
	R52I	ORF3a	Arg^521^→Ile
	D3L	ORF3a	Asp^3^→Leu
	S235F	Nucleoprotein	Ser^235^→Phe
	SGF3675–3677	ORF1a	Deletion mutation
	Y73C	ORF3a	Tyr^73^→Cys
	D614G	Spike glycoprotein	Asp^614^→Gly

P.1	L18F	Spike glycoprotein	Leu^18^→Phe
	T20N	Spike glycoprotein	Thr^20^→Asn
	P26S	Spike glycoprotein	Pro^26^→Ser
	D138Y	Spike glycoprotein	Asp^138^→Tyr
	R190S	Spike glycoprotein	Arg^190^→Ser
	K417T	Spike glycoprotein	Lys^417^→Thr
	H655Y	Spike glycoprotein	His^655^→Tyr
	N501Y	Spike glycoprotein	Asn^501^→Tyr
	E484K	Spike glycoprotein	Glu^484^→Lys
	T1027I	Spike glycoprotein	Thr^1027^→Ile
	E92K	ORF3a	Glu^92^→Lys
	P80R	Nucleoprotein	Pro^80^→Arg
	S1188L	ORF1a	Ser^1188^→Leu
	K1795Q	ORF1a	Lys^1795^→Gln
	SGF3675–3677	ORF1a	Deletion mutation
	E5665D	ORF1b	Glu^5665^→Asp
	D614G	Spike glycoprotein	Asp^614^→Gly

B.1.351	L18F	Spike glycoprotein	Leu^18^→Phe
	D80A	Spike glycoprotein	Asp^80^→Ala
	D215G	Spike glycoprotein	Asp^215^→Gly
	R246I	Spike glycoprotein	Arg^2461^→Ile
	K417N	Spike glycoprotein	Lys^417^→Asn
	E484K	Spike glycoprotein	Glu^484^→Lys
	N501Y	Spike glycoprotein	Asn^501^→Tyr
	A701V	Spike glycoprotein	Ala^701^→Val
	P71L	Envelope protein	Pro^71^→Leu
	T205I	Nucleoprotein	Thr^205^→Ile
	K1655N	ORF1a	Lys^1655^→Asn
	SGF3675–3677	ORF1a	Deletion mutation
	D614G	Spike glycoprotein	Asp^614^→Gly

B.1.617.2	T19R	Spike glycoprotein	Thr^19^→Arg
	G142D	Spike glycoprotein	Gly^142^→Asp
	156del	Spike glycoprotein	Deletion mutation
	R158G	Spike glycoprotein	Arg^158^→Gly
	L452R	Spike glycoprotein	Leu^452^→Arg
	T478K	Spike glycoprotein	Thr^478^→Lys
	D614G	Spike glycoprotein	Asp^614^→Gly
	P681R	Spike glycoprotein	Pro^681^→Arg
	D950N	Spike glycoprotein	Asp^950^→Asn
	157del	Spike glycoprotein	Deletion mutation

**TABLE 3:** Mutational landscape of different emerging variants of interest

Variant name	Mutation	Mutation position	Amino acid change
B.1.525	Q677H	Spike glycoprotein	Gln^677^→His
	A67V	Spike glycoprotein	Ala^67^→Val
	E484K	Spike glycoprotein	Glu^484^→Lys
	D614G	Spike glycoprotein	Asp^614^→Gly
	F888L	Spike glycoprotein	Phe^888^→Leu
	T2007I	ORF1a	Thr^2007^→Ile
	P314F	ORF1b	Pro^314^→Phe
	I82T	Membrane protein	Ile^82^→Thr
	A12G	Nucleoprotein	Ala^12^→Gly
	T205I	Nucleoprotein	Thr^205^→Ile
	R81C	5′ UTR	Arg^81^→Cys

B.1.526	L5F	Spike glycoprotein	Leu^5^→Phe
	T95I	Spike glycoprotein	Thr^95^→Ile
	D253G	Spike glycoprotein	Asp^253^→Gly
	E484K	Spike glycoprotein	Glu^484^→Lys
	D614G	Spike glycoprotein	Asp^614^→Gly
	A701V	Spike glycoprotein	Ala^701^→Val
	L3201P	ORF1a	Leu^3201^→Pro
	T265I	ORF1a	Thr^265^→Ile
	P314L	ORF1b	Pro^314^→Leu
	Q1011H	ORF1b	Gln^1011^→His
	P42L	ORF3a	Pro^42^→Leu
	Q57H	ORF3a	Gln^57^→His
	T11I	ORF8	Thr^11^→Ile
	R81C	5′ UTR	Arg^81^→Cys

P.2	E484K	Spike glycoprotein	Glu^484^→Lys
	D614G	Spike glycoprotein	Asp^614^→Gly
	V1176F	Spike glycoprotein	Val^1176^→Phe
	L3468V	ORF1a	Leu^3468^→Val
	L3930F	ORF1a	Leu^3930^→Phe
	P314L	ORF1b	Pro^314^→Leu
	A119S	Nucleoprotein	Ala^119^→Ser
	R203K	Nucleoprotein	Arg^203^→Lys
	G204R	Nucleoprotein	Gly^204^→Arg
	M234I	Nucleoprotein	Met^234^→Ile
	R81C	5′ UTR	Arg^81^→Cys

B.1.427/B.1.429	L452R	Spike glycoprotein	Leu^452^→Arg
	D614G	Spike glycoprotein	Asp^614^→Gly

**FIG 19:**
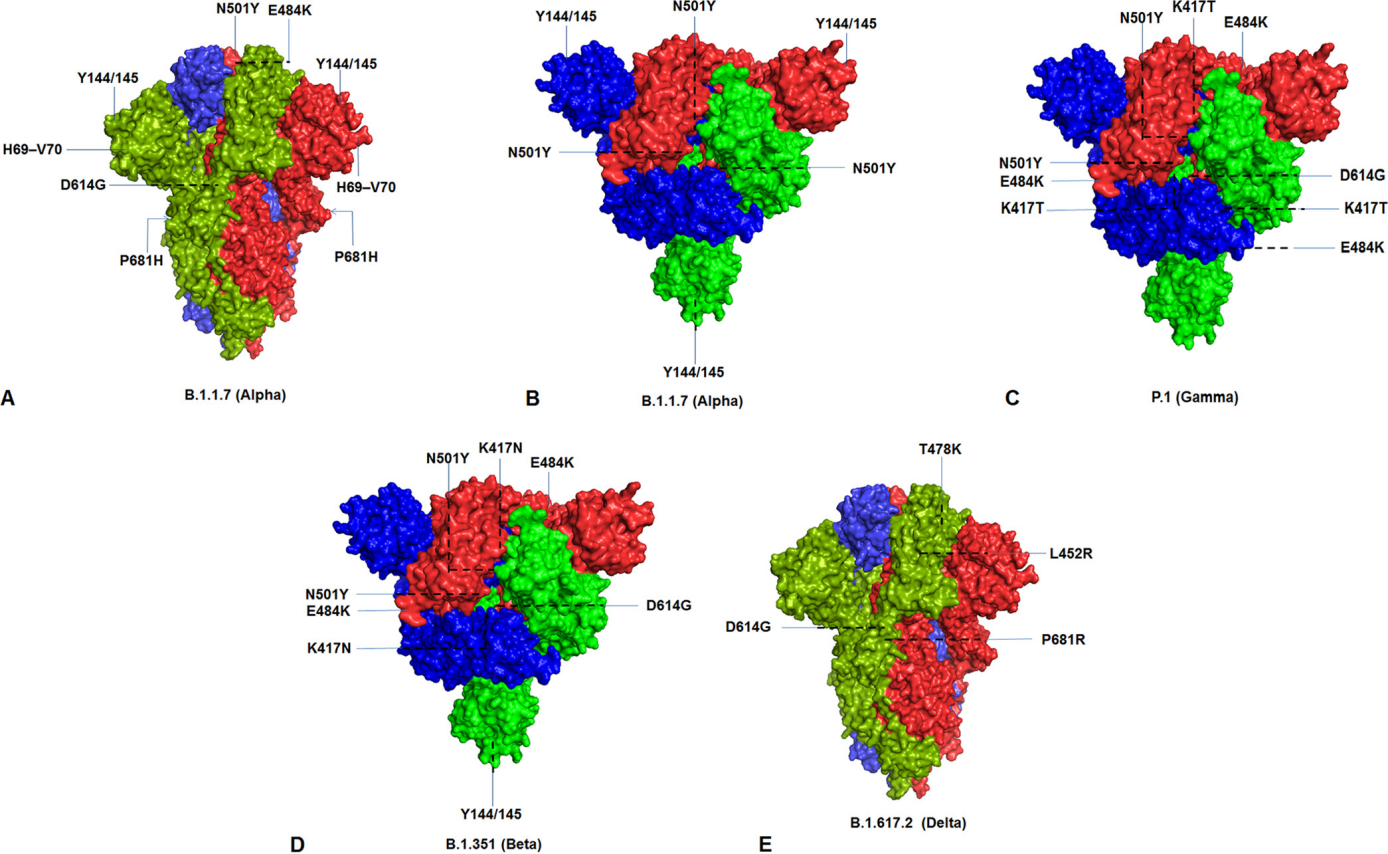
Significant mutational landscapes in spike protein of significant VOCs between December 2019 and June 2021. (A) Mutational landscape in the spike protein of the B.1.1.7 (Alpha) lineage (closed form). (B) Mutational landscape in the spike protein of the B.1.1.7 (Alpha) lineage (closed form with 90° rotation) (C) Mutational landscape in the spike protein of the P.1 (Gamma) lineage. (D) Mutational landscape in the spike protein of the B.1.351 (Beta) lineage. (E) Mutational landscape in the spike protein of the B.1.617.2 (Delta) lineage.

**FIG 20:**
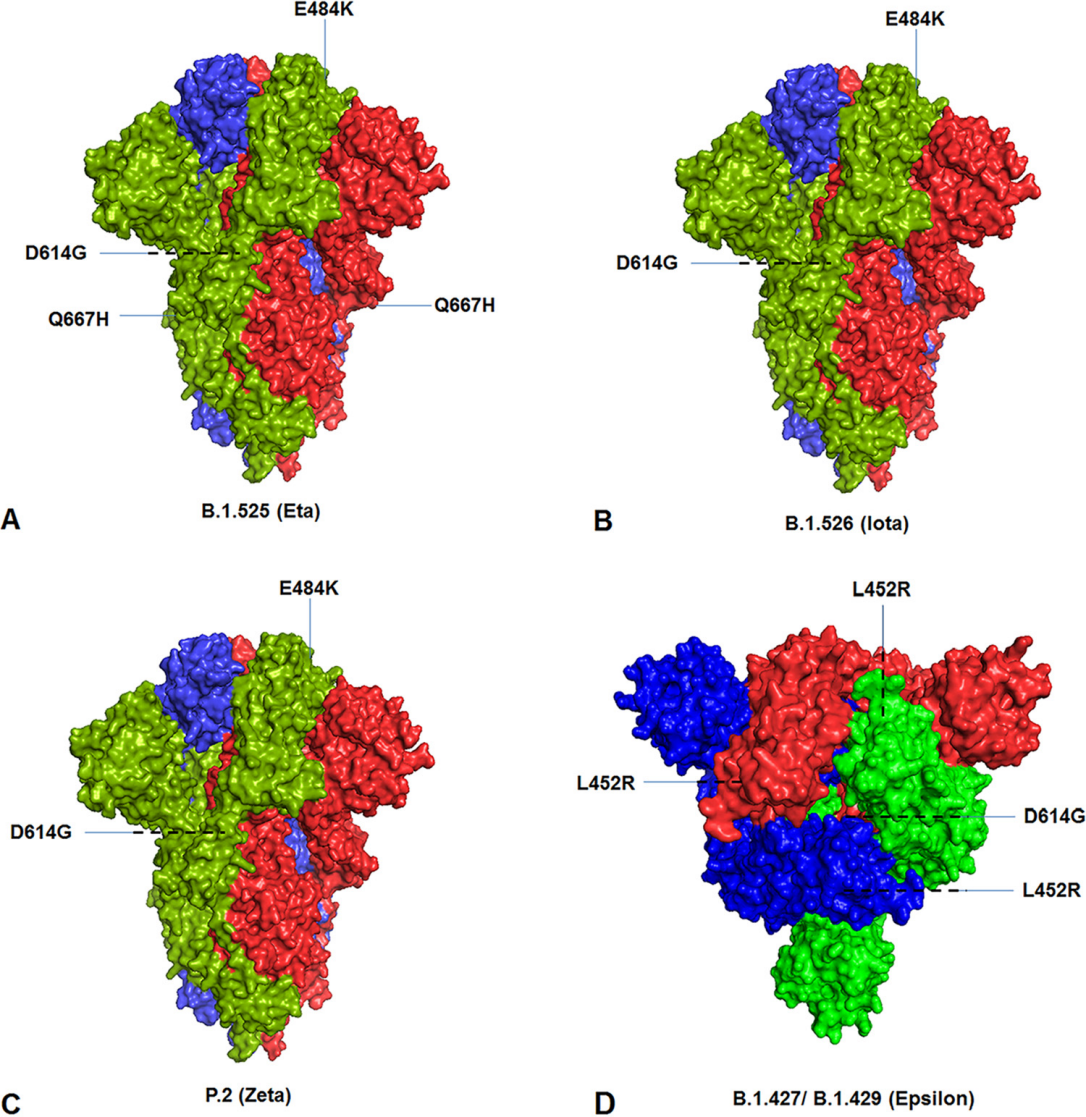
Significant mutational landscapes in spike protein of significant VOIs between December 2019 and June 2021. (A) Mutational landscape in the spike protein of the B.1.525 (Eta) lineage. (B) Mutational landscape in the spike protein of the B.1.526 (Iota) lineage. (C) Mutational landscape in spike protein of the P.2 (Zeta) lineage. (D) Mutational landscape in the spike protein of the B.1.427/B.1.429 (Epsilon) lineage.

The significant mutational positions in the S-protein are illustrated through the diagrammatic representation of lineages B.1.1.7 (Fig. 19A and B), P.1 (Fig. 19C), B.1.351 (Fig. 19D), B.1.617.2 (Fig. 19E), B.1.525 (Fig. 20A), B.1.526 (Fig. 20B), P.2 (Fig. 20C), and B.1.427/B.1.429 (Fig. 20D).

It has been reported that there are three significant mutations in the P.1 lineage. These are present in spike receptor binding domain (RBD) (E484K, K417T/N, and N501Y) (7, 18). Simultaneously, it was reported that the mutation D614G can augment the capability to spread compared to the wild type (10, 18). Another mutation (nonsynonymous mutation P681H) was observed in the S protein of the B.1.1.7 lineage.

### Some significant mutations (E484K, K417T/N, N501Y, and D614G) found in emerging variants and their structural landscapes.

**FIG 21:**
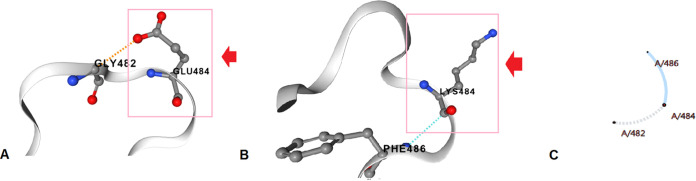
Structural landscape of the E484K mutation. (A) Contacts of the wild type (Glu). (B) Interactions of the mutant type (Lys). (C) Interactions of the wild type (Glu) with the mutant (Lys).

We performed structural landscape analysis of some significant mutations, such as E484K, K417T/N, N501Y, and D614G, which are frequently reported in emerging variants. We have analyzed the E484K mutation. Here, the structure of E484K changes due to the replacement Glu^484^→Lys. The structural analysis of E484K is shown in different forms, such as the interaction abilities of the wild-type residues (Fig. 21A) and the interaction abilities of the mutant protein structure (Fig. 21B), and in addition, a snapshot during the toggle of the molecular interaction is included to understand the interactions between the wild-type (dashed lines) and mutant (straight lines) residues (Fig. 21C). The wild-type amino acid configuration shows the interaction of Glu^484^ with Gly^482^. In contrast, the mutant amino acid configuration shows the interaction of Lys^484^ with Phe^486^.

**FIG 22:**
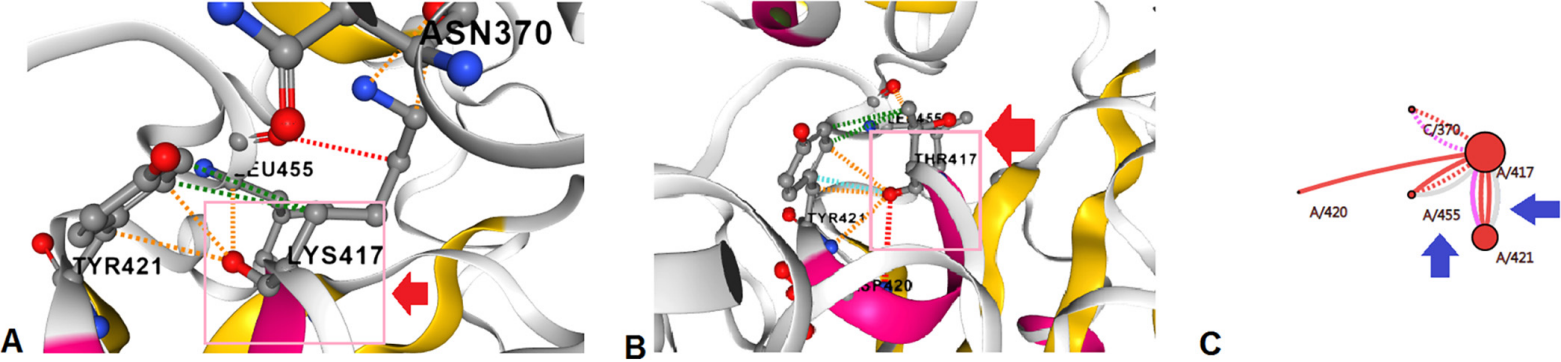
Structural landscape of the K417T mutation. (A) Contacts of the wild type (Lys). (B) Interactions of the mutant type (Thr). (C) Interactions of the wild type (Lys) with the mutant (Thr).

We next analyzed the K417T mutation, and the structure of K417T is altered due to the replacement Lys^417^→Thr. The structural analysis of K417T is shown in different forms, such as interaction abilities of the wild-type residues (Fig. 22A), interaction abilities of the mutant residues (Fig. 22B), and interactions of wild-type with the mutant residues (Fig. 22C). The wild-type amino acid configuration shows the interaction of Lys^417^ with Leu^455^, Tyr^421^, and Asn^370^. In contrast, the mutant amino acid configuration shows the interaction of Thr^417^ with Tyr^421^, Leu^455^, and Asp^420^.

**FIG 23:**
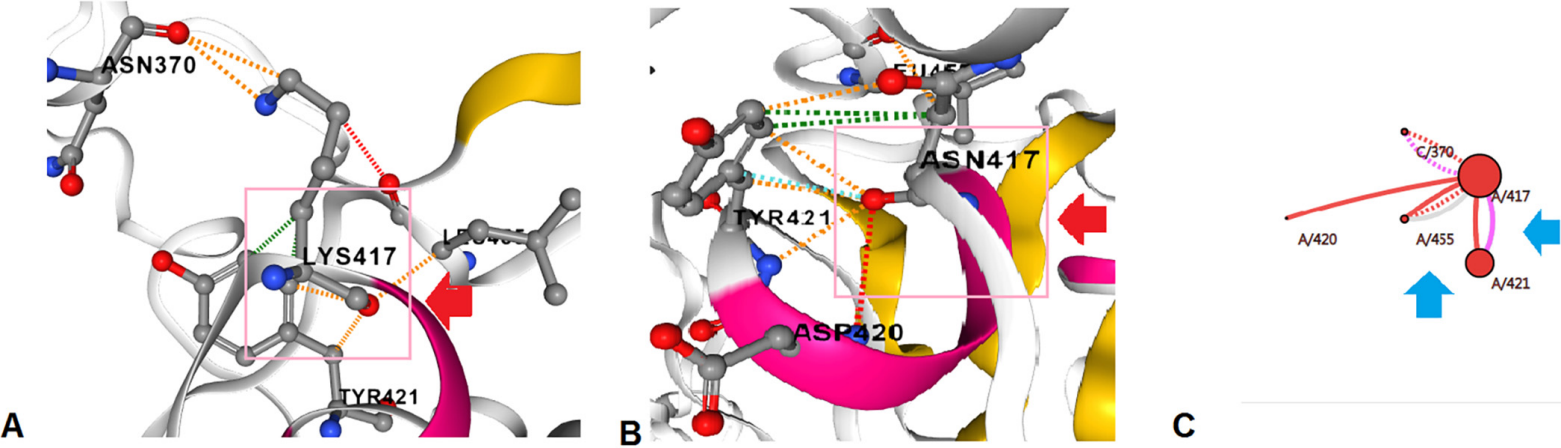
Structural landscape of the K417N mutation. (A) Contacts of the wild type (Lys). (B) Interactions of the mutant (Asn). (C) Interactions of the wild type (Lys) with the mutant (Asn).

Furthermore, we analyzed the K417N mutation. Here, the structure of K417N is changed due to the replacement Lys^417^→Asn. The structural analysis of K417N is shown in different forms, such as interaction abilities of the wild-type residues (Fig. 23A), interaction abilities of the mutant residues (Fig. 23B), and interactions of the wild-type and mutant residues (Fig. 23C). The wild-type amino acid shows the interaction of Lys^417^ with Leu^455^, Tyr^421^, and Asn^370^. In contrast, the mutant amino acid configuration shows the interaction of Asn^417^ with Tyr^421^, Leu^455^, and Asp^420^.

**FIG 24:**
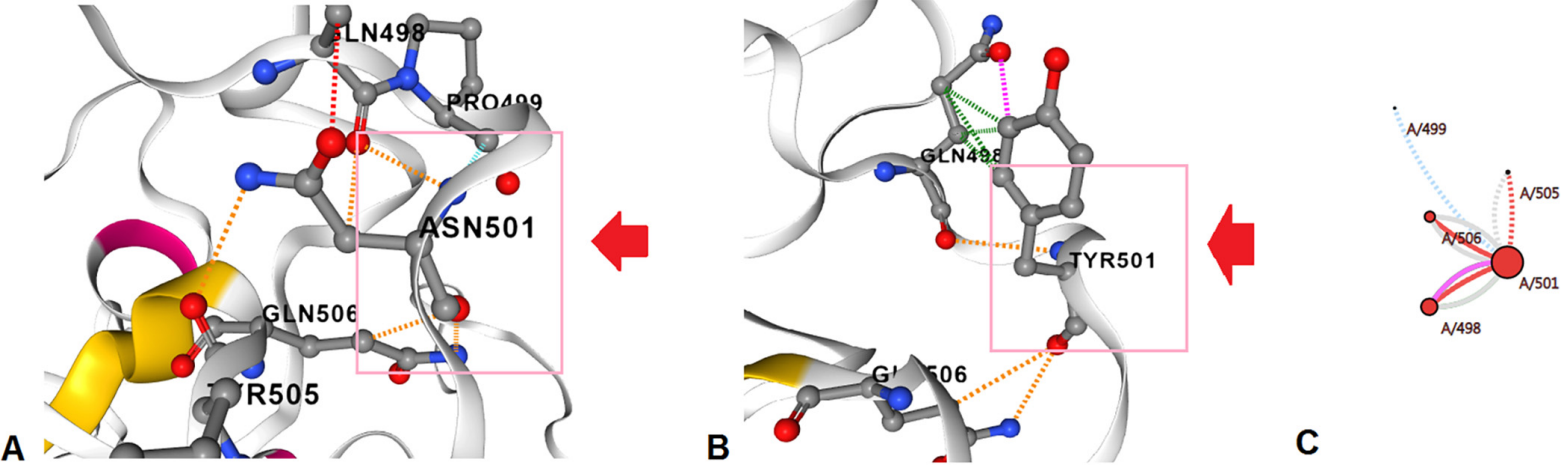
Structural landscape of the N501Y mutation. (A) Contacts of the wild type (Asn). (B) Interactions of the mutant (Tyr). (C) Interactions of the wild type (Asn) with the mutant (Tyr).

Simultaneously, we also analyzed the N501Y mutation. Here, the structure of N501Y was altered due to the replacement Asn^501^→Tyr. The structural analysis of N501Y is shown in different forms, such as interaction abilities of the wild-type residues (Fig. 24A), interaction abilities of the mutant residues (Fig. 24B), and interaction of the wild-type and mutant residues (Fig. 24C). The wild-type amino acid configuration shows the interaction of Asn^501^ with Gln^506^ and Pro^499^. In contrast, the mutant amino acid configuration shows the interaction of Tyr^501^ with Gln^498^ and Gln^506^. The wild-type and mutant residues also show some other interactions.

**FIG 25:**
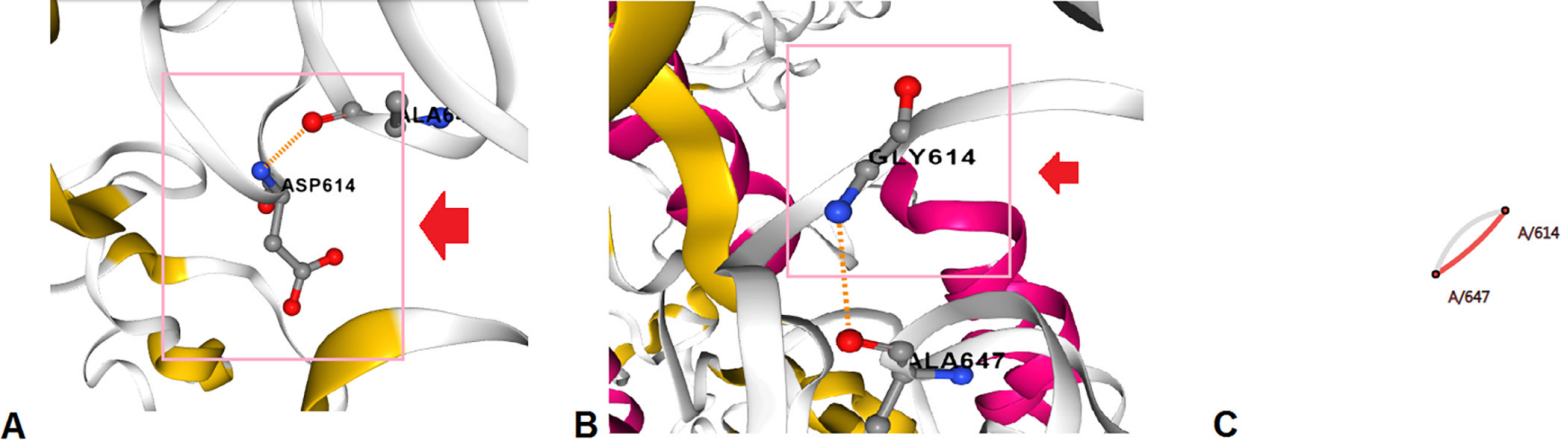
Structural landscape of the D614G mutation. (A) Contacts of the wild type (Asp). (B) Interactions of the mutant (Gly). (C) Interactions of the wild type (Asp) with the mutant (Gly).

Finally, the D614G mutation was analyzed. Here, the structure of D614G was changed due to the replacement of Asp^614^→Gly. The structural analysis of D614G is shown in different forms, such as interaction abilities of the wild-type residues (Fig. 25A), interaction abilities of the mutant residues (Fig. 25B), and interactions of wild-type and mutant residues (Fig. 25C). The wild-type amino acid configuration shows the interaction of Asp^614^ with Ala^647^. In contrast, the mutant amino acid configuration shows the interaction of Gly^614^ with Ala^647^. The interaction pattern between the residues of the wild type and mutant did not show any changes.

### The new SARS-CoV-2 variants and efficacy of vaccines against these variants.

We have tried to understand the new SARS-CoV-2 variants and their recognition by neutralizing antibodies produced by different vaccines. The data were obtained from the various available data in the literature (Table 4). The effectiveness of vaccines against newly emerging SARS-CoV-2 variants is noted from different reports in the literature and is summarized in Table 5. These two tables help us to evaluate the reduction in vaccine efficacy by the newly developed virus variants.

**TABLE 4:** Major approved COVID-19 vaccines and their reported efficacy against SARS-CoV-2

Sl no.	Vaccine	Efficacy of COVID-19 vaccine against SARS-CoV-2 (%)	Reference
1	Ad26.CoV2.S (Johnson & Johnson)	85	35
2	BNT162b2 mRNA COVID-19 (Pfizer)	95	36
3	mRNA-1273 (Moderna)	94.1	37
4	Sputnik V COVID-19 (Gamaleya)	91.6	38
5	AZD1222 COVID-19 (AstraZeneca)	70.4	39
6	NVX-CoV2373 (Novavax)	96.4	40
7	CoronaVac COVID-19 (Sinovac)	79	41
8	BBIBP-CorV (Sinopharm)	79	12

**TABLE 5:** Efficacy of some approved significant vaccines against new SARS-CoV-2 variants

Sl no.	New significant SARS-CoV-2 lineage	SARS-CoV-2 vaccine efficacy^*a*^			
		Ad26.COV2.S (Johnson & Johnson)	BNT162b2 (Pfizer)	mRNA-1273 (Moderna)	NVX-CoV2373 (Novavax)
1	B.1.1.7	NA	Reduced neutralizing activity	Decreased neutralizing antibodies	85.6% efficacy in United Kingdom population; 60% efficacy in South African population
2	P.1	NA	Significant reduction in neutralization efficacy	Decreased neutralizing antibodies	NA
3	B.1.351	64.0% efficacy in Brazilian population; 52% efficacy in South African population	Reduced neutralizing activity	Reduced neutralizing activity	49% efficacy in South African population

a For details, see references 14, 17, 18, and 42. NA, not available.

## DISCUSSION

The first section of the data analysis has shown a depiction of the current scenario of the evolution of emerging variants of SARS-CoV-2 virus, the cluster of genome samples and their divergence, and the geographical distribution and transmission pattern of the variants. Presently, the transmission of the virus has become uncontrolled in different parts of the world. According to published reports, some varieties have shown higher transmission potential, such as the B.1.1.7 lineage (19). Similarly, we have found a higher transmission pattern in the B.1.1.7 lineage. Therefore, the transmission pattern analyzed for these variants is highly significant in the present perspective when the transmission continues to increase in several countries. We have also described the circulating frequencies of all the lineages and all the newly emerging lineages. From the analysis, we found that 100% frequency was achieved by lineage B.1.1.7 in early October 2020. We also found entropy diversity and mutational event diversity throughout the genomes of all newly emerging lines.

We have critically evaluated the mutational landscape of all the above emerging variants. The data analysis has shown significant mutations throughout the genomes of all newly emerging lineages. The important mutations were recorded in the spike protein, especially in the RBD of all newly emerging lineages. There is already recorded evidence that the mutations in spike protein, especially in the RBD, can influence the transmission pattern of this virus (10). We have also evaluated the structural landscape of several significant mutations (E484K, K417T/N, N501Y, and D614G) in the emerging variants. The structural analysis will help us understand the structural contacts of the wild-type protein, structural connections of the mutant protein, and wild-type and mutant interactions, assisting in understanding the mutational landscape. These significant mutations are related to more infectivity in most emerging lineages and death tolls (7, 10, 18).

It is a known fact that the E484K mutation affects neutralization by convalescent-phase sera or monoclonal antibodies (MAbs) (19, 20). Similarly, the combination mutation of K417N and N501Y affects neutralization by MAbs and convalescent-phase sera (19–21). Therefore, our analysis confirms this observed phenomenon and is very noteworthy for now. Finally, we analyzed the vaccines’ efficacy against the new variants of this virus and compared them with the vaccines’ reported effectiveness. We found a reduction in vaccine efficacy for the new variants. However, the efficacy of all approved vaccines has not been analyzed against all newly emerging SARS-CoV-2 variants. Therefore, further evaluation is urgently needed.

Furthermore, the year 2021 will be more challenging due to the emergence of numerous variants. Therefore, we raise the following queries. How will the recently emerged variants affect people's health? Will the COVID-19 vaccines protect people against the recently emerged variants? Can the COVID-19 vaccination program be successfully implemented across the developing world?

### Conclusion.

The emergence of several new lineages of SARS-CoV-2 due to viral mutation is crucial as vaccination programs have started throughout the globe. Scientists have started research to protect against all new significant mutant variants. They have already designed next-generation vaccines against this pandemic virus using different epitopes from all important mutant variants, including the Wuhan variant (22). However, our analysis will help to design future countrywide pandemic planning, focusing on emerging variants, as well as next-generation vaccine development using alternative wild-type antigens and significant viral antigens, and immediate planning for ongoing vaccination programs worldwide.

## MATERIALS AND METHODS

### Data collection.

We retrieved or collected different data sets for new SARS-CoV-2 variants from the WHO (23) and CDC (24). We searched for different keywords in databases such as Web of Science (25), PubMed (26, 27), and Google Scholar. For the database search, we used keywords such as “SARS-CoV-2 variants,” “variants of consequence,” “VOI,” “VOC,” and “variants and vaccines,” etc. We also searched for the new variants with keywords such as “B.1.1.7 lineage,” “P.1 lineage,” and “B.1.351 lineage.” A search for two other variant names with the keywords “B.1.427/B.1.429 variant” and “B.1.617.2 variant” was also performed. Similarly, we searched for three other different variant names as keywords: “B.1.526 variant,” “B.1.525 variant,” and “P.2 variant.”

We have tried to collect data from different sources, such as The New York Times (coronavirus-variant-tracker) (28) and various other resources. For further data collection, we used several databases and servers, such as Nextstrain (SARS-CoV-2 resources) (28, 29), Pango lineages (30), Pango lineages on GitHub (31), and COVID-3D (32, 33). Nextstrain uses the data from GISAID. We analyzed and retrieved the data from the Nextstrain server in April 2021.

We have followed the nomenclature for lineages of this virus proposed by Rambaut et al. (34).

### Data analysis and interpretation.

For data analysis, we used several servers, such as Nextstrain (SARS-CoV-2 resources) (26, 27), Pango lineages (30), Pango lineages on GitHub (31), and COVID-3D (32). We also used COVID-3D for the structural analysis of significant mutations (E484K, K417T/N, N501Y, and D614G) in emerging variants (18). We have depicted the study methodology through a flowchart summarizing the overall process in Fig. 1.
